# Comparative mRNA and miRNA expression in European mouflon (*Ovis musimon*) and sheep (*Ovis aries*) provides novel insights into the genetic mechanisms for female reproductive success

**DOI:** 10.1038/s41437-018-0090-1

**Published:** 2018-05-21

**Authors:** Ji Yang, Xin Li, Yin-Hong Cao, Kisun Pokharel, Xiao-Ju Hu, Ze-Hui Chen, Song-Song Xu, Jaana Peippo, Mervi Honkatukia, Juha Kantanen, Meng-Hua Li

**Affiliations:** 1 CAS Key Laboratory of Animal Ecology and Conservation Biology, Institute of Zoology, Chinese Academy of Sciences (CAS), Beijing, 100101 China; 2 College of Life Sciences, University of Chinese Academy of Sciences (UCAS), Beijing, 100049 China; 3 Production Systems, Natural Resources Institute Finland (Luke), 31600 Jokioinen, Finland

**Keywords:** Evolutionary biology, Molecular evolution, Gene expression

## Abstract

Prolific breeds of domestic sheep (*Ovis aries*) are important genetic resources due to their reproductive performance, which is characterized by multiple lambs per birth and out-of-season breeding. However, the lack of a comprehensive understanding of the genetic mechanisms underlying the important reproductive traits, particularly from the evolutionary genomics perspective, has impeded the efficient advancement of sheep breeding. Here, for the first time, by performing RNA-sequencing we built a de novo transcriptome assembly of ovarian and endometrial tissues in European mouflon (*Ovis musimon*) and performed an mRNA–miRNA integrated expression profiling analysis of the wild species and a highly prolific domestic sheep breed, the Finnsheep. We identified several novel genes with differentially expressed mRNAs (e.g., *EREG*, *INHBA*, *SPP1*, *AMH*, *TDRD5*, and *ZP2*) between the wild and domestic sheep, which are functionally involved in oocyte and follicle development and fertilization, and are significantly (adjusted *P*-value < 0.05) enriched in the Gene Ontology (GO) terms of various reproductive process, including the regulation of fertilization, oogenesis, ovarian follicle development, and sperm–egg recognition. Additionally, we characterized 58 differentially expressed miRNAs and 210 associated target genes that are essential for the regulation of female reproduction cycles through specific regulatory networks [e.g., (*miR-136*, *miR-374a*, *miR-9-5p*)-(*EREG*, *INHBA*)]. Furthermore, our integrated mRNA and miRNA expression profiling analysis elucidated novel direct and indirect miRNA/mRNA causal regulatory relationships related to the reproductive traits of the *Ovis* species. This study provides in-depth insights into the genomic evolution underlying the reproductive traits of the *Ovis* species and valuable resources for ovine genomics.

## Introduction

Reproduction is an important but complex biological process in mammals. From the physiological perspective, the reproductive performance of a female is attributable to a series of biological events occurring between oocyte maturation and birth (Vinet et al. 2012). An increased number of offspring is largely associated with increased ovulation rates during the estrous cycle and subsequent successful embryonic and fetal development (Vinet et al. 2012; Warriach et al. 2015). The ovary and uterus are two essential parts of the female reproductive tract. Function of the ovary includes the production of oocytes, excretion of reproductive hormones, and maintenance of female estrus cycles (Peters and McNatty 1980). The endometrium, which is the inner-most epithelial layer of the mammalian uterus, functions to prevent adhesions between the opposing walls of the myometrium, thereby maintaining the patency of the uterine cavity (Fazleabas and Strakova 2002). Therefore, the ovary, endometrium, and their communications can directly affect female reproductive success (Peters and McNatty 1980; Fazleabas and Strakova 2002; Wira et al. 2015). From the evolutionary perspective, the reproductive success of females is optimally balanced by the frequency of the estrous cycle per year, time between parturition and first ovulation, number and size of offspring, and time at which sexual maturity is reached (Walker et al. 2008; Bronson 2009; Barrett et al. 2009).

During and after domestication, livestock reproductive traits have been continuously selected. Wild sheep such as Asian mouflon *Ovis orientalis* and European mouflon *Ovis musimon* typically show pronounced seasonal estrous in all reproductive parameters and are typically monotonous (Lincoln 1989; Santiago-Morenoa et al. 2000; Garel et al. 2005). After domestication, many sheep (*Ovis aries*) breeds continue to maintain seasonal estrus and have one lamb per litter, while several prolific breeds typically demonstrate the capability of non-seasonal breeding and have multiple lambs per litter, which could be ascribed to long-term artificial and natural selection (Rasali et al. 2006). Additionally, prolific breeds of domestic sheep show much earlier sexual maturation (as early as 6 months age) than wild sheep (more than 1.5 years age; Cugnasse et al. 1985; Garel et al. 2005). Early genetic mapping studies have detected a few quantitative trait loci (QTLs) and functional genes (e.g., *BMPR1B*, *BMP15*, and *GDF9*) associated with high prolificacy in various sheep breeds (Hanrahan et al. 2004; Bodin et al. 2007; Abdoli et al. 2013). However, to our knowledge no study has explored the genetic mechanisms underlying the development of reproductive traits from wild to domestic sheep.

Transcriptome profiling using next-generation sequencing has been successfully used to measure the levels of transcripts and their isoforms (Wang et al. 2009). In livestock, mRNA profiling has been used to characterize large-scale gene expression patterns and identify differentially expressed genes that are specifically associated with production traits (Mortazavi et al. 2008; Jiang et al. 2014; Suravajhala et al. 2016; Clark et al. 2017). Additionally, microRNA (miRNA) profiling has successfully used to identify particular miRNAs, predict their target genes, and analyze their potential biological functions (Ambros 2004; Bartel 2009; McDaneld 2009; Liu et al. 2014). In sheep, previous RNA profiling investigations have revealed novel mRNAs, miRNAs, relevant functional genes, and variants associated with a variety of traits, including high prolificacy traits such as multiple births and non-seasonal breeding (Di et al. 2014; Pokharel et al. 2015; Hu et al. 2016). However, integrated analyses of large-scale mRNA and miRNA expression profiling in two (e.g., ovary and endometrium) or more female reproductive tissues from sheep are scarce (but see Miao and Qin 2015; Pokharel et al. 2018). Additionally, to date no full-length transcriptome has been available for any wild sheep, which has impeded studies investigating the genomic evolution and functional diversification of specific miRNA genes (Liu et al. 2008; Berezikov 2011; Ameres and Zamore 2013) between wild and domestic sheep.

In this study, we performed genome-wide profiling of the mRNA and miRNA in ovarian and endometrial tissues in European mouflon and a prolific breed of domestic sheep, the Finnsheep (Fig. S1 and Table S1). In particular, for the first time we reconstructed a de novo transcriptome of wild sheep (Fig. S2 and Table S1) using the Trinity platform, which provides a novel algorithm for assembling full-length transcripts (Grabherr et al. 2011; Haas et al. 2013). The European mouflon is considered one of the close wild relatives to domestic sheep (Hiendleder et al. 2002; Lv et al. 2015). The Finnsheep, which is a native sheep breed from Finland, is well-known worldwide due to its excellent prolific performance, including its early maturity (at c. 6 months of age), multiple lamb births (typically 3–5 lambs per birth, and the recorded highest number of lambs is 9), and non-seasonal breeding (Maijala 1996; Li et al. 2011; Mullen and Hanrahan 2014). Nevertheless, early mRNA and miRNA studies investigating the prolificacy traits in Finnsheep have included very limited sample sizes, and the analyses of mRNAs and miRNAs were performed separately (Pokharel et al. 2015; Hu et al. 2016). By performing integrated and comparative analyses of the data within and between species, our aims are threefold: (i) to reveal the genetic mechanisms underlying the evolution of the reproduction traits in species of the genus *Ovis*; (ii) to decipher the genetic architecture of the high prolificacy traits in a domestic sheep breed; and (iii) to create novel mRNA and miRNA genomic resources associated with reproductive traits in wild and domestic sheep species. Our results provide a comprehensive understanding of the genetic regulatory network responsible for the high prolificacy traits and molecular mechanisms underlying their evolution in *Ovis* species, which could also be applied to other mammals.

## Materials and methods

### Sample collection, RNA isolation, and sequencing

All animal handling was carried out under the license approved by the Southern Finland Animal Experiment Committee (approval No. ESAVI/5027/04.10.03/2012). From the Finnsheep, six ovarian samples (F1-O, F2-O, F3-O, F4-O, F5-O, and F6-O) were collected from six ewes (F1, F2, F3, F4, F5, and F6), and two endometrial samples (F2-E and F3-E) were taken from F2 and F3 individuals. The ewes in the experiment were mature, with an approximate age and weight of 4.25 years and 71.2 kg, respectively. The ovarian samples were collected at the follicular growth phase based on hormonal profiles, and endometrial samples were collected during early pregnant stage after ovulation (Pokharel et al. 2018). Three European mouflon ewes (M1, M2, and M3) were killed by hunters in autumn of 2013 (M1 and M2) and 2014 (M3). To obtain comparable sample sizes between the wild and domestic sheep, we collected six ovarian samples (M1-OA, M1-OB, M2-OA, M2-OB, M3-OA, and M3-OB) representing two replicates (coded as -OA and -OB) from each of the three European mouflons. Two endometrial samples (M2-EA and M2-EB) were taken by cytobrush from both uterine horns of the older individual M2 (5–6 years old), and we did not take endometrial samples from the other two younger wild sheep. We did not have any information about the stage of the cycle in the mouflons except that the older one (M2) had a corpus luteum. In total, 16 samples consisting of 12 ovarian samples and 4 endometrial samples were included in the mRNA profiling analysis. Additionally, all 16 samples were included in the miRNA profiling analysis, except for M2-EA (Table S1).

After the sample collection, the tissue samples of domestic sheep were quickly stored in RNAlater reagent (Ambion/Qiagen, Valencia, CA, USA) per the manufacturer’s instructions and transported to the laboratory. For the European mouflons, the collected samples were stored in RNAlater within 1 h of death due to the forest areas. The tissue samples were then transferred to be stored at −80°C in laboratory until extraction. We used an RNeasy plus mini kit (Qiagen, Valencia, CA, USA) to extract the mRNA and miRNA from the tissues according to the manufacturer’s protocol. The RNA concentration and RNA integrity number were measured using a Bioanalyzer 2100 (Agilent Technologies, Waldbronn, Germany). Libraries of mRNAs and miRNAs were prepared using the Illumina’s TruSeq library preparation kits and sequenced using Illumina Hiseq2000 at the Finnish Microarray and Sequencing Center, Turku, Finland. The mRNA libraries were sequenced using 100 base-pair (bp) paired-end sequencing chemistry, whereas the miRNAs were sequenced using the single-end 50 bp approach.

### Quality control and mapping

FastQC v.0.11.4 (http://www.bioinformatics.babraham.ac.uk/projects/fastqc/) was used to examine the quality of the raw RNA-Seq (both the mRNA-Seq and miRNA-Seq) reads, and the adapter sequences were removed using the program Cutadapt v.1.9.1 (Martin 2011). The obtained trimmed mRNA-Seq reads were then mapped against the sheep reference genome (Oar v.4.0) supported by the gene annotation file of Ensembl release 83 using the program TopHat v.2.0.8b (Trapnell et al. 2012). For the miRNA-Seq data, the program PRINSEQ-LITE v.0.20.4 (Schmieder and Edwards 2011) was used for further miRNA read preprocessing (including filtering for reads containing ambiguous reads, data reformatting, and adapter trimming). Clean reads between 18 and 26 nucleotides (nt) in length were used for the subsequent analysis. Then, the Bowtie-build tool was used to build an index for the genome alignment using the sheep genome assembly Oar v.3.1 (ftp://ftp.ensembl.org/pub/release-83/fasta/ovis_aries/dna/), and the clean reads (18–26 nt) were mapped to the index using the program Bowtie v.2.2.1 (Langmead et al. 2009). The bioinformatics pipeline used to analyze the mRNA-Seq and miRNA-Seq data is summarized in Fig. S1.

### De novo transcriptome assembly and annotation of European mouflon

We used the Trinity v.2.1.1 package (Grabherr et al. 2011) to construct a de novo transcriptome assembly of European mouflon using the ovarian (M1-OA, M1-OB, M2-OA, M2-OB, M3-OA, and M3-OB) and endometrial (M2-EA and M2-EB) mRNA sequences. The quality of the de novo assembly was assessed by examining the proportion of reads mapped to the assembly. We used the RSEM (RNA-Seq by expectation-maximization) tool (Li and Dewey 2011) to estimate the number of RNA-Seq fragments that map to each contig in the individual M2 (M2-OA, M2-OB, M2-EA, and M2-EB), and detected the differentially expressed genes between the two types of tissues, i.e., ovary and endometrium, using the EdgeR package (Robinson et al. 2010). Unigenes that had |log_2_(fold change)| ≥ 2 and a false discovery rate ≤ 0.01 were considered significantly differentially expressed. The differentially expressed unigenes were mapped to the NCBI nt collection database using the BlastN (Altschul et al. 1997) tool in the Blast v.2.4.0+ package (http://blast.ncbi.nlm.nih.gov) with a matched standard “*e*-value = 1e-10 and matching ratio = 100%”. The program PANTHER v.10 (Mi et al. 2016) was used to perform the Gene Ontology (GO) and Kyoto Encyclopedia of Genes and Genomes (KEGG) analyses, and the threshold was set to a *P*-value < 0.05 after the Bonferroni correction. The bioinformatics analysis workflow is summarized in Fig. S2.

### mRNA differential expression and functional analysis

After the mapping, the programs Cufflinks, Cuffmerge, and Cuffdiff in the Cufflinks software package v.2.1.1 (Trapnell et al. 2012) were used to assemble the mRNA clean reads and calculate the gene expression levels based on the fragments per kilobase of exon per million fragments mapped (FPKM) values. First, we performed the mRNA transcript assembly and created a “merged transcripts.gtf” for all ovarian or endometrial samples from one species using the programs Cufflinks and Cuffmerge. Then, the program Cuffdiff was used to calculate the level of gene expression and perform differential expression analyses between the two species (i.e., ovary/endometrium: European mouflon vs. Finnsheep) or two types of tissues (i.e., European mouflon: ovary vs. endometrium). The differentially expressed genes (DEGs) were defined as genes that showed |log_2_(fold change)| ≥ 2 and *P*_adj_ (i.e. adjusted *P*-value) ≤ 0.01 in the comparisons. Furthermore, we performed GO and KEGG pathway enrichment analyses of functional genes associated with the differentially expressed mRNAs using the Database for Annotation, Visualization, and Integrated Discovery (DAVID) tools v.6.8 (Huang et al. 2009). The enriched GO terms were considered significant at an adjusted *P*-value < 0.05. A word cloud was created for the GO terms and pathways using an online Wordcloud generator (https://www.jasondavies.com/wordcloud/).

### miRNA quantification and differential expression analysis

The miRDeep2 v.0.0.7 software package (Friedländer et al. 2012) was used to predict the putative precursor miRNA sequences, and precursor miRNAs with 10 or fewer reads and miRDeep2 scores < 5 were removed. Then, the filtered miRNAs were aligned to the miRNA database miRBase v.21 (http://www.mirbase.org/) to identify mature miRNAs that contain sheep miRNAs (i.e., miRNAs deposited in the miRBase of *O. aries*), conserved miRNAs (i.e., miRNAs matching those in other mammals in miRBase with fewer than four mismatches), and novel miRNAs (remaining miRNAs).

The differentially expressed ovarian and endometrial miRNAs (including the sheep and conserved miRNAs) between European mouflon and Finnsheep and between the two types of tissues (ovary vs. endometrium) in European mouflon were identified using the DESeq v.1.22.0 program (Anders and Huber 2010). By applying the threshold values of *P*_adj_ (i.e., adjusted *P*-value) ≤ 0.05 and |log_2_(fold change)| ≥ 1, the remaining miRNAs considered to be upregulated or downregulated in one of the two species or types of tissues were used in the following mRNA–miRNA integrated analyses.

### miRNA–mRNA regulatory network analysis

The program TargetScan v.7.0 (Agarwal et al. 2015) was used to predict the binding of the differentially expressed miRNAs to their putative targets, i.e., the 5′ seed regions (2–8 nt) of the miRNAs complementary to the conserved 8- and 7-mer sites in the 3′-untranslated region of the mRNA (Lewis et al. 2005). miRNAs typically function in post-transcriptional regulation by suppressing or silencing specific target genes. Thus, we searched for inverse correlations between the expression levels of the miRNAs and their predicted target genes, i.e., between the upregulated miRNAs and downregulated mRNAs, and vice versa. To narrow the list of potential miRNA–target interactions, the putative target genes were overlapped with the DEGs in the mRNA profiling data. Then, target genes with a context++ score percentile lower than 50 were removed. Furthermore, the upregulated and downregulated miRNAs and their target genes were included in the network visualization analysis using the program Cytoscape v.3.4.0 (Shannon et al. 2003). Only the top 10 downregulated miRNAs were considered core miRNAs and used to build the main regulatory network. Furthermore, GO and pathway enrichment analyses of the miRNA target genes were conducted using DAVID tools v.6.8 (Huang et al. 2009). GO terms and pathways with an adjusted *P*-value < 0.05 were considered significantly enriched. A flowchart of the mRNA–miRNA integrated analysis is provided in Fig. S1.

To confirm the credibility of the inferred miRNA and target gene pairs, we searched the miRTarBase (http://mirtarbase.mbc.nctu.edu.tw/; Chou et al. 2016) database and previous studies for differentially expressed miRNAs, predicted target genes, and miRNA–gene pairs consistent with those identified in this study.

## Results and Discussion

### mRNA and miRNA profiling

In total, we generated approximately 127.1 Gb mRNA and 21.3 Gb miRNA raw data. After filtering, we obtained a large number of high-quality reads: 1083.6 million mRNA and 56.8 million miRNA reads in European mouflon, and 934.6 million mRNA and 127.8 million miRNA reads in Finnsheep. Most high-quality mRNA and miRNAs have been mapped to the *O*. *aries* reference genome Oar_v.4.0 (Table S2). Regarding the mRNA expression, more than 89% of the genes were expressed at <50 FPKM and only <1.1% of the genes were expressed at >500 FPKM in all four tissue-species combinations (Table S3). Altogether, 23 557 common genes were expressed in the ovaries and 19 208 common genes were expressed in the endometria (Table S3).

In the de novo assembly of the European mouflon transcriptome, 1083.6 million clean mRNA reads were assembled into 844 129 unigenes, with an N50 length of 700 bp and an average length of 562 bp (Table 1). The observed length of N50 is comparable to that reported in domestic sheep (e.g., 508–1482 bp) (Yue et al. 2015; Zhang et al. 2015), thus highlighting the high quality of the assembled European mouflon transcriptome. Based on the transcriptome, we identified 5739, 5831, 5897, and 5981 annotated DEGs in the four comparisons between the ovary and endometrium tissues (M2-OA vs. M2-EA, M2-OA vs. M2-EB, M2-OB vs. M2-EA, and M2-OB vs. M2-EB) in European mouflon, respectively. However, when the analyses were performed using the sheep reference genome (Oar v.4.0), we found far fewer DEGs (e.g., 1925 DEGs for M2-O-B vs. M2-E-B). Despite the possible implication of using the *O. aries* genome as a reference, the observation suggests that the de novo approach is accurate and that the quality of the de novo assembly is good.

**Table 1:** Summary of constituent data of Trinity-assembled European mouflon transcriptome

Description	Number
Before trimming	
Raw reads	1 083 877 020
After trimming	
Clean reads	1 083 600 777
Average length of clean reads (bp)	101
N percentage	0
GC content (%)	48.92
After assembly	
Total Trinity “genes”	844 129
Total Trinity transcripts	1 102 841
Statistics based on all transcript contigs	
Mean length of contigs (bp)	947.39
N50 (bp) of contigs	2169
Statistics based on unigenes	
Mean length of unigenes (bp)	562.14
N50 (bp) of unigenes	700

We identified 373 mature miRNAs in the ovaries and 192 mature miRNAs in the endometria, including 89 and 59 sheep miRNAs, 174 and 104 conserved miRNAs, and 110 and 29 novel miRNAs in the two tissues, respectively (Table S4). High levels of expression were observed in the sheep miRNAs, followed by conserved miRNAs and novel miRNAs (Table S4). Of the 122 novel miRNAs (Table S5), 20 miRNAs were also identified as novel miRNAs and validated in our previous study (Hu et al. 2016). The chromosomal distribution of the sheep and conserved miRNAs indicated the presence of large numbers of miRNAs on chromosomes 18 and X, whereas none of the miRNAs were expressed on chromosome 8 based on the ovarian data and chromosomes 8, 17, and 25 based on the endometrium data (Table S6).

### mRNAs and miRNAs showing the greatest expression

**Fig. 1:**
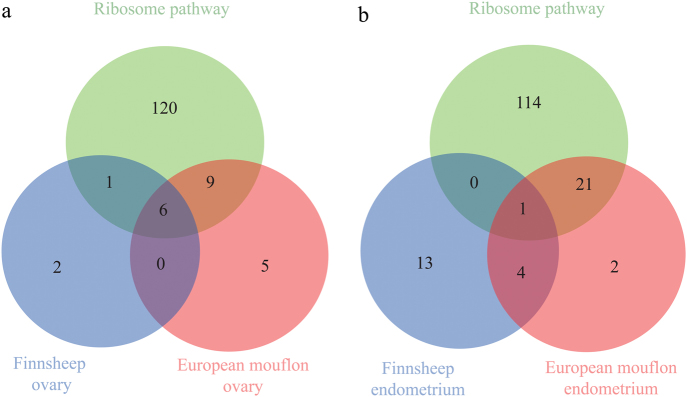
Number of genes exhibiting the greatest expression (FPKM > 3000) in the Finnsheep and European mouflon ovaries (**a**) and endometria (**b**), and their overlap with genes in the ribosome pathway

Of the genes exhibiting the greatest expression (FPKM > 3000) in ovary, 7 genes in the Finnsheep and 15 genes in the European mouflon encode ribosomal protein (Fig. 1a and Table S7). In the endometrium, only 1 top gene identified in the Finnsheep and 22 top genes identified in the European mouflon encode ribosomal protein involved in the ribosome pathway (Fig. 1b and Table S7). The ribosome pathway has been reported to be among the most significant pathways involved in early embryogenesis (Berres et al. 2017). The observed large number of ribosome family genes in both tissues of European mouflon could be one explanation for the low reproductive success of the wild sheep (i.e., seasonal estrus, 1.5 years of age to sexual maturation and one lamb per litter; Cugnasse et al. 1985; Garel et al. 2005) compared to the prolificacy of domestic sheep, including Finnsheep (i.e., out-of-season breeding, 6 months of age to sexual maturation and three lambs per litter on average; Maijala 1996; Li et al. 2011; Mullen and Hanrahan 2014). Moreover, the observed smaller number of ribosome family genes expressed at a higher level in Finnsheep than in European mouflon is likely due to the evolution of reproductive traits between the two species during the domestication and long-term artificial selection processes. Additionally, this observation could be related to the environment of the mouflon being harsher than the domestic environment of the Finnsheep; in such a harsh environment, it may be beneficial to have fewer offspring.

Of the 10 most highly expressed ovarian miRNAs, 7 (*miR-10b*, *miR-143*, *miR-26a*, *miR-27b*, *let-7f*, *miR-191*, and *miR-22-3p*) miRNAs are common between European mouflon and Finnsheep (Table S8). Of the endometrial miRNAs, three (*miR-10b*, *miR-100*, and *miR-34c-5p*) miRNAs are highly expressed in both species (Table S8). We also observed tissue- and species-specific miRNAs (Table S9), which could possibly be associated with particular functions. For instance, *miR-181a*, which is specifically and abundantly expressed in the Finnsheep ovary, was demonstrated to be associated with pregnancy complications and was more highly expressed in normal term than in pre-term delivery (Mayor-Lynn et al. 2011). Additionally, *miR-186*, which is specifically and abundantly expressed in the European mouflon endometrium, was observed to be more highly expressed in endometrial cancer (Zhou et al. 2008).

### Differentially expressed mRNAs and miRNAs

**Fig. 2:**
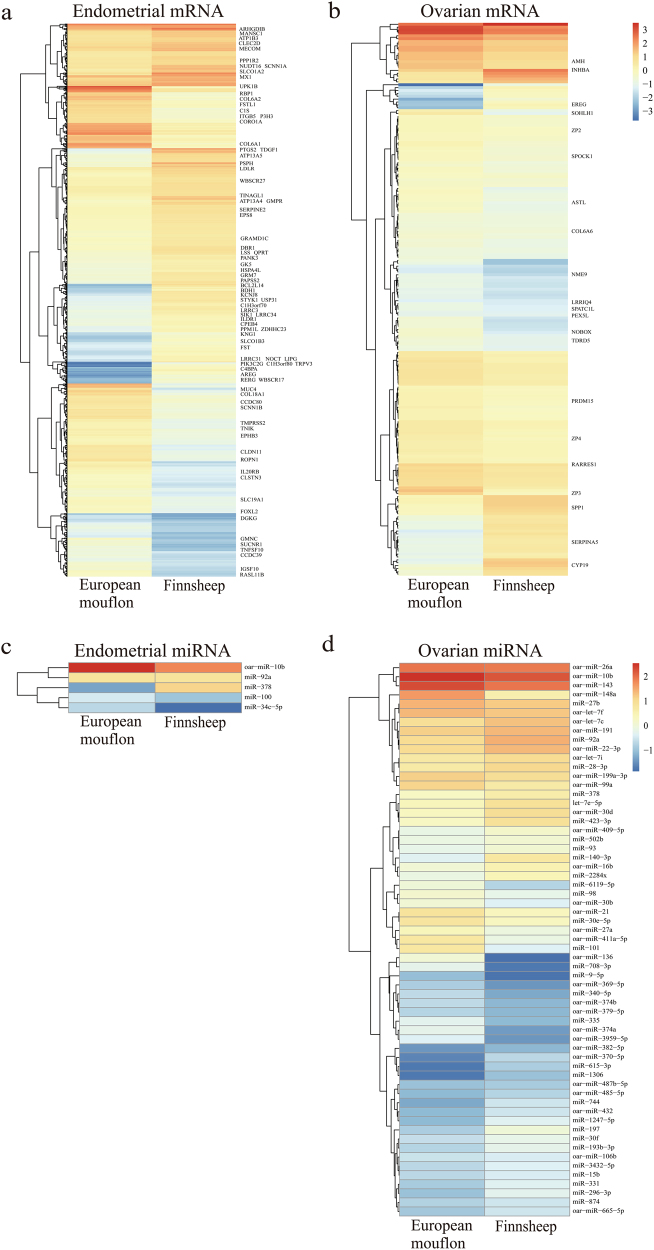
Heat maps of differentially expressed mRNAs and miRNAs between Finnsheep and European mouflon. **a** Heat map showing the expression intensity of 1065 differentially expressed genes (DEGs) based on a comparison of endometria from the two species. **b** Heat map showing the expression intensity of 192 DEGs based on a comparison of ovaries from the two species. **c** Heat map showing the expression intensity of 5 differentially expressed miRNAs based on a comparison of endometria from the two species. **d** Heat map showing the expression intensity of 53 differentially expressed miRNAs based on a comparison of ovaries from the two species. Heat maps were generated using the R heat map package v.1.0.8 (http://cran.r-project.org/web/packages/pheatmap/), which is based on the *k*-means clustering algorithm and the Boolean values to determine if the rows (i.e., mRNAs or miRNAs) should be clustered. The important genes associated with the reproductive traits such as early mature, fecundity, non-seasonal breeding, and total lambs born are labeled in the figure

In total, 192 and 1065 DEGs responsible for ovarian and endometrial mRNAs were identified between the two species, respectively. Of these DEGs, 149 ovarian and 492 endometrial DEGs were upregulated in European mouflon, while 43 ovarian and 573 endometrial DEGs were upregulated in Finnsheep (Tables S10 and S11). Additionally, we identified 53 and 5 differentially expressed ovarian and endometrial miRNAs between the two species, respectively (Table 2). Based on the differentially expressed mRNAs and miRNAs in the tissues, heat maps were constructed (Fig. 2) and showed distinct expression patterns between European mouflon and Finnsheep, indicating that the evolution of the gene- and post-transcriptional regulation could have contributed to the improved reproductive performance during the domestication process.

**Table 2:** List of significantly differentially expressed miRNAs between Finnsheep and European mouflon tissues (ovary and endometrium)

miRNA	log_2_(fold change)^a^	*P*_adj_^b^	miRNA	log_2_(fold change)^a^	*P*_adj_^b^
**Ovary (European mouflon up or Finnsheep down)**			**Ovary (European mouflon down or Finnsheep up)**		
miR-136	7.70	2.03E-40	miR-140-3p	4.22	2.60E-30
miR-708-3p	6.01	1.40E-08	miR-370-5p	3.95	1.88E-13
miR-101	4.62	4.22E-05	miR-615-3p	3.87	3.09E-08
miR-374a	4.54	1.52E-15	miR-197	3.50	3.28E-03
miR-148a	4.47	1.07E-14	miR-1306	3.36	9.62E-14
miR-3959-5p	4.06	1.19E-16	miR-1247-5p	3.20	5.09E-03
miR-411a-5p	3.56	9.57E-27	miR-744	3.14	2.94E-11
miR-6119-5p	3.10	6.12E-03	miR-296-3p	2.94	1.75E-17
miR-335	3.03	4.52E-03	miR-432	2.54	1.10E-04
miR-9-5p	2.73	8.40E-03	miR-30d	2.54	1.92E-22
miR-369-5p	2.45	3.26E-05	miR-423-3p	2.39	3.17E-13
miR-340-5p	2.41	1.24E-09	miR-331	2.28	8.14E-03
miR-21	2.24	9.93E-20	miR-2284x	2.24	1.59E-13
miR-27a	2.13	3.16E-03	miR-193b-3p	1.98	6.84E-03
miR-30e-5p	2.07	9.30E-11	miR-16b	1.81	1.97E-12
miR-143	1.99	8.11E-03	miR-665-5p	1.71	1.60E-04
miR-99a	1.96	8.07E-26	miR-485-5p	1.67	1.62E-03
miR-374b	1.90	2.14E-03	miR-30f	1.65	5.96E-05
miR-10b	1.81	8.11E-03	let-7e-5p	1.64	2.69E-07
miR-30b	1.71	3.39E-02	miR-382-5p	1.47	9.02E-05
miR-379-5p	1.38	1.64E-09	miR-92a	1.39	5.07E-08
miR-98	1.38	1.45E-02	miR-28-3p	1.39	2.15E-05
miR-27b	1.20	7.73E-05	miR-378	1.31	7.16E-03
			miR-409-5p	1.28	2.55E-03
			miR-15b	1.26	1.27E-05
			miR-3432-5p	1.20	3.95E-02
			let-7c	1.19	1.40E-08
			miR-93	1.18	3.06E-04
			miR-22-3p	1.17	5.07E-08
			miR-874	1.08	2.00E-02
**Endometrium (European mouflon up or Finnsheep down)**			**Endometrium (European mouflon down or Finnsheep up)**		
miR-34c-5p	4.73	9.99E-03	miR-378	3.18	2.84E-02
miR-10b	3.82	4.92E-07			
miR-100	3.29	2.12E-03			
miR-92a	2.18	2.39E-02			

^a^The threshold for fold change is set to log_2_(fold change) > 1 or <−1

^b^The statistical significance is evaluated as *P*_adj_ (i.e., adjusted *P*-value) < 0.05

Of the upregulated genes in the ovary and endometrium tissues from the Finnsheep, 4 genes (*CYP19*, *PTGS2*, *FST*, and *PAPSS2*) are associated with early maturity, 14 genes (*EREG*, *INHBA*, *SERPINA5*, *SPP1*, *LIPG*, *PTGS2*, *AREG*, *TDGF1*, *FST*, *C4BPA*, *SERPINE2*, *TRPV3*, *LDLR*, and *TINAGL1*) are associated with fecundity, and two genes (*CYP19* and *SERPINE2*) are associated with non-seasonal breeding. In addition, we compared the 192 ovarian DEGs with the QTLs for the reproductive traits in the Sheep QTL database (https://www.animalgenome.org/cgi-bin/QTLdb/OA/), and found 8 genes (*NME9*, *LRRIQ4*, *SPATC1L*, *PRDM15*, *PEX5L*, *COL6A6*, *RARRES1*, and *SPOCK1*; Table S12) overlapped with previously identified QTLs associated with traits such as reproductive seasonality and total lambs born. Similarly, we found 73 endometrial DEGs that overlapped with the reported QTLs for the reproductive seasonality, and 1 gene, i.e., *RASL11B*, was overlapped with the QTL chromosome 6 as follows: 68295117–68445447 for the total lambs born trait (Table S13).

### GO and KEGG enrichments of differentially expressed mRNAs

**Fig. 3:**
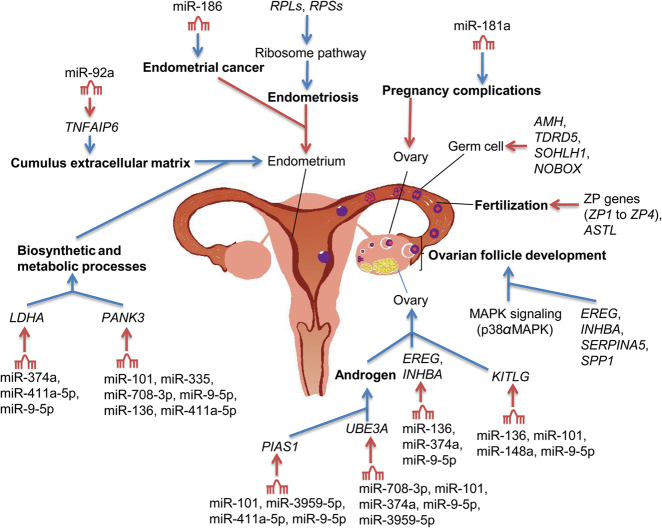
Functional roles of the main mRNAs and miRNAs in the endometrium and ovary of *Ovis* species. Ovarian and endometrial network of differentially expressed mRNAs and top 10 Finnsheep downregulated (i.e., European mouflon upregulated) miRNAs and target genes are shown, along with their functional roles associated with reproduction (bold type). Regulation and alteration of these functional roles are responsible for the evolution of high prolificacy traits. Red and blue arrows indicate downregulation and upregulation of the reproductive organs, respectively

In the functional enrichment analysis of the 192 ovarian DEGs between European mouflon and Finnsheep, the top enriched GO categories were mainly relevant to reproduction, including fertilization, reproductive developmental process, reproductive process, oogenesis, and ovarian follicle development (Fig. S3a and Table S14). Fertilization refers to the fusion of gametes that initiates the development of a new individual organism (Merriam-Webster 2017). During the process, the *ZP* proteins (*ZP1* to *ZP4*) in the zona pellucida function to surround the egg, bind the sperm and mediate species-selective sperm–oocyte interactions (Suzuki et al. 2015; Avella et al. 2016). *ZP2* peptide beads have been shown to markedly inhibit the fertilization of ovulated eggs, prevent sperm binding in mice and humans (Gupta et al. 2012; Avella et al. 2016) and induce infertility in female mice (Avella et al. 2016). *ASTL*, which is involved in the negative regulation of fertilization by the *ZP2* and *ZP4* genes, plays a definitive role in ensuring monospermic fertilization by encoding ovastacin, which is a cortical granule protease (Burkart et al. 2012). Thus, the significantly upregulated expression of the *ASTL*, *ZP2*, *ZP3*, and *ZP4* genes in the European mouflon ovary (Table S10) may indicate that these genes play a role in the low fertility of wild sheep compared to the high prolificacy of domestic sheep (Fig. 3).

The GO terms of the reproductive development process and ovarian follicle development included the genes *EREG*, *INHBA*, *SERPINA5*, and *SPP1*, which are significantly upregulated in Finnsheep (Tables S10 and S14). *EREG* is a member of the epidermal growth factor family, which plays an important role in mediating the luteinizing hormone-induced ovulation process in preovulatory follicles (Amsterdam 2010; Kim et al. 2011). *INHBA* plays an essential role in early oocyte and follicular development (Thomas et al. 2003) and has a significant effect on the litter size in sheep (Leyhe et al. 1994) and goat (Zi et al. 2012). *SERPINA5*, which encodes a serine protease inhibitor, may be involved in follicular growth and atresia in the bovine ovarian follicle (Hayashi et al. 2011). *SPP1* is located on sheep chromosome 6 close to the high prolificacy gene *FecB* in sheep (Montgomery et al. 1993; Montgomery et al. 1994). Additionally, the genes *AMH*, *TDRD5*, *SOHLH1*, and *NOBOX* are upregulated in European mouflon (Tables S10 and S14). *AMH* is a central influencing factor in gonad development, and could inhibit germ cell proliferation and differentiation in both sexes (Durlinger et al. 2002). Recent clinical data have indicated that serum *AMH* level is a useful indicator of ovarian aging and premature ovarian failure in women (de Vet et al. 2002; Alipour et al. 2015). The *TDRD5* protein is an evolutionarily conserved protein involved in germ cell development. Injection of *TDRD5*-deficient round spermatids into oocytes result in fertile offspring (Yabuta et al. 2011). *SOHLH1* is a critical regulator of oogenesis and can downregulate *NOBOX*, which functions to disrupt folliculogenesis (Pangas et al. 2006). Therefore, our results suggest that the upregulated expression of these genes (i.e., *EREG*, *INHBA*, *SERPINA5*, and *SPP1* in Finnsheep, and *AMH*, *SOHLH1*, *TDRD5*, and *NOBOX* in European mouflon) is most likely associated with the hyper-prolificacy of domestic sheep (e.g., Finnsheep) and the single lambing of wild sheep (e.g., European mouflon; Fig. 3). We also observed other top GO categories related to immune processes such as the humoral immune response and humoral immune response mediated by circulating immunoglobulin (Table S14). The KEGG analysis of the 192 ovarian DEGs identified three significant (adjusted *P*-value < 0.05) pathways, i.e., ovarian steroidogenesis, complement and coagulation cascades, and MAPK signaling pathways (Table S14). The MAPK (i.e., p38αMAPK) pathway has been shown to play an essential role in female reproduction, particularly in follicular development (Hu et al. 2017) (Fig. 3).

In the functional enrichment of the endometrial DEGs between the wild and domestic sheep, the GO terms of the 492 upregulated genes (*RARRES2*, *LTBP1*, and *TGFB3*) in European mouflon were most significantly enriched in the developmental process, such as anatomical structure development (Fig. S3b and Table 3), whereas the top enriched GO terms of the 573 upregulated genes (*PTGS2*, *LDLR*, and *SREBF1*) in Finnsheep included biosynthetic and metabolic processes, such as lipid metabolic process (Fig. S3b and Table 3). An early study investigating the bovine endometrial transcriptome indicated an earlier shift from proliferation to metabolism at early diestrus in the cow (Mesquita et al. 2015). Cell proliferation was enriched in the small follicle endometrium, whereas biosynthetic and metabolic processes were enriched in the large follicle endometrium, which presented higher proliferative activity in the luminal epithelium, glandular epithelium, and stroma than in the small follicle endometrium (Mesquita et al. 2015). Additionally, the large follicle endometrium is an active endometrial phenotype that is associated with the optimal uterine environment (Miller and Moore 1976; Mesquita et al. 2015). Thus, Finnsheep could have a larger follicle endometrium and better receptivity of the uterus than European mouflon (Fig. 3).

**Table 3:** The top 10 significantly enriched biological process GO terms associated with the upregulated genes in Finnsheep and European mouflon endometrial mRNAs

GO term	GO number	Cluster genes	Total genes	Percentage (%)	*P*-value
Finnsheep^a^					
Single-organism metabolic process	GO:0044710	146	577	25.30	7.77E-16
Small-molecule metabolic process	GO:0044281	79	577	13.69	1.40E-10
Single-organism process	GO:0044699	344	577	59.62	6.42E-09
Lipid metabolic process	GO:0006629	57	577	9.88	9.24E-09
Secondary alcohol biosynthetic process	GO:1902653	10	577	1.73	1.50E-08
Sterol biosynthetic process	GO:0016126	10	577	1.73	2.55E-08
Single-organism biosynthetic process	GO:0044711	55	577	9.53	2.66E-08
Small-molecule biosynthetic process	GO:0044283	30	577	5.20	3.07E-08
Coenzyme metabolic process	GO:0006732	23	577	3.99	1.44E-07
Cholesterol biosynthetic process	GO:0006695	9	577	1.56	1.86E-07
European mouflon^a^					
System development	GO:0048731	147	498	29.52	5.42E-14
Single-multicellular organism process	GO:0044707	178	498	35.74	1.79E-13
Multicellular organism development	GO:0007275	153	498	30.72	4.32E-13
Multicellular organismal process	GO:0032501	187	498	37.55	2.79E-12
Animal organ development	GO:0048513	115	498	23.09	1.23E-11
Anatomical structure development	GO:0048856	162	498	32.53	2.92E-11
Anatomical structure morphogenesis	GO:0009653	103	498	20.68	3.25E-11
Single-organism developmental process	GO:0044767	161	498	32.33	7.03E-11
Developmental process	GO:0032502	162	498	32.53	2.24E-10
Cardiovascular system development	GO:0072358	52	498	10.44	4.36E-10

^a^We performed GO annotations for European mouflon and Finnsheep’s upregulated genes in endometrium to understand the gene evolution underlying the endometrium’s evolution

In the KEGG analysis of the 1065 endometrial DEGs, the top enriched pathways were enriched in the extracellular matrix (ECM)–receptor interaction, steroid biosynthesis, and biosynthetic and metabolic processes (Table S15). The ECM, which is a complex matrix of biological macromolecules, can transfer signals to cells via surface receptors during cell adhesion and other various signaling transduction pathways. Additionally, the ECM can send signals to the cytoplasm and nucleus to influence gene expression or cellular activities (Alldinger et al. 2006). Thus, adhesion and connection mechanisms could play important roles in the endometrium at the molecular level, and different cell adhesion may occur between wild and domestic sheep. In addition, the differentially expressed mRNAs between the ovarian and endometrial tissues from the European mouflon and their functional analysis based on the reference genome and de novo approaches are presented in the Supplementary Results and Tables S16–S18.

### Post-transcriptional regulatory network of miRNAs and target genes

**Fig. 4:**
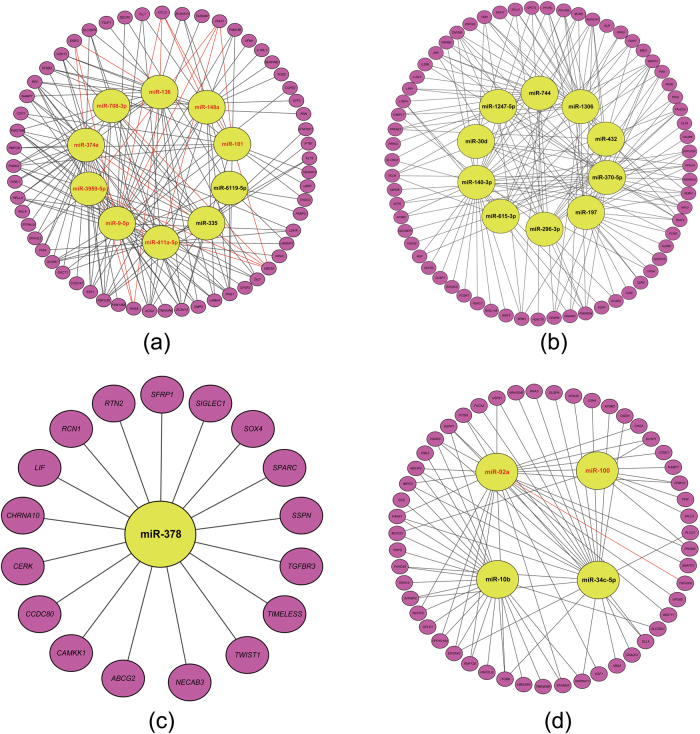
Main miRNA–gene regulatory networks. **a** Ovarian network of top 10 Finnsheep downregulated (i.e., European mouflon upregulated) miRNAs and 57 target genes. **b** Ovarian network of top 10 European mouflon downregulated (i.e., Finnsheep upregulated) miRNAs and 64 target genes. **c** Endometrial network of 1 Finnsheep upregulated (i.e., European mouflon downregulated) miRNAs and 17 target genes. **d** Endometrial network of 4 Finnsheep downregulated (i.e., European mouflon upregulated) miRNAs and 51 target genes. Large and small ellipses represent miRNAs and their target genes, respectively. The important miRNAs, genes, and miRNA–gene pairs discussed in the text are marked with red color

We obtained 100, 64, 17, and 51 target genes for the up- and downregulated miRNAs in particular tissues (Table 4). In the main downregulated network of the Finnsheep ovary, which comprised 10 downregulated miRNAs and 57 upregulated target genes (Fig. 4a), several important miRNAs and functional genes were involved in the reproductive process. For instance, *miR-136*, which exhibited the largest fold change (Table 2), has been shown to be downregulated in term labor women compared with that in pre-term labor women by real-time quantitative reverse transcription-PCR (qRT-PCR) (Montenegro et al. 2009). The genes *EREG* and *INHBA*, which are co-regulated by three miRNAs (*miR-136*, *miR-374a*, and *miR-9-5p*), were identified as important genes responsible for the hyper-prolificacy of Finnsheep in the above GO enrichment analysis of ovarian mRNAs (Table 2). Additionally, four miRNAs (*miR-101*, *miR-3959-5p*, *miR-411a-5p*, and *miR-9-5p*) targeted *PIAS1* and five miRNAs (*miR-708-3p*, *miR-101*, *miR-374a*, *miR-3959-5p*, and *miR-9-5p*) targeted *UBE3A*. Both genes modulate androgen receptor (AR)-dependent transactivation (Nishida and Yasuda 2002; Ramamoorthy and Nawaz 2008). AR is well-known for its roles in male reproduction and has been recently revealed to be essential for normal female fertility, including follicle health, development, and ovulation (Walters et al. 2010). Moreover, four miRNAs (*miR-136*, *miR-101*, *miR-148a*, and *miR-9-5p*) co-regulated *KITLG*, which is a strong candidate gene related to litter size in goats (An et al. 2015).

**Table 4:** The number of predicted target genes for differentially expressed (DE) miRNAs and their overlapping with the DE genes from mRNA profile

Tissue	DE miRNAs^a^	Predicted target genes by TargetScan	DE genes from mRNA profile^a^	Overlapped genes
Ovary	30 (Finn up or Mou down)	1265	1000	100
	23 (Mou up or Finn down)	1130	721	64
Endometrium	1 (Finn up or Mou down)	242	1174	17
	4 (Mou up or Finn down)	627	1258	51

*Finn* Finnsheep, *Mou* European mouflon

^a^The thresholds for identifying DE miRNAs and selecting DE genes are log_2_(fold change) > 1 or <−1 and *P*_adj_ (i.e., adjusted *P*-value) < 0.05

The main downregulated network of the European mouflon ovary comprises 10 downregulated miRNAs that co-regulate 64 upregulated target genes (Fig. 4b). In the network, *miR-370-5p*, which exhibited the second largest fold change (Table 2), was reported to be differentially expressed between the ovaries of sheep producing single lamb and two lambs, and plays a vital role in sheep follicular development and litter size regulation (Qi et al. 2015). Additionally, *miR-1306* exhibits a lower expression level in the ovaries of monotocous goats than that in ovaries of polytocous goats (An et al. 2016).

Regarding the endometrial networks, Finnsheep had 1 upregulated miRNA with 17 downregulated target genes and 4 downregulated miRNAs with 51 upregulated target genes, while European mouflon had inverse regulation patterns (Fig. 4c, d). In the upregulated network of European mouflon (Fig. 4d), *miR-92a* targeted the gene *TNFAIP6*, which is a key catalyst in the formation of the cumulus extracellular matrix and is indispensable for female fertility (Fülöp et al. 2003). Additionally, *miR-100* was observed to be highly expressed in monotocous goats (An et al. 2016). In addition, other identified miRNAs and target genes (Figs. S4 and S5) may also influence the reproductive traits of European mouflon and Finnsheep (see Supplementary Results).

In the GO enrichment analysis of the genes that were targeted by the differentially expressed ovarian and endometrial miRNAs between Finnsheep and European mouflon (Supplementary Results and Tables S19–S22), the 64 upregulated genes targeted by 23 downregulated miRNAs in the ovary of Finnsheep were significantly enriched in the GO categories of the reproductive process and regulation of developmental process (Table S19). Altogether, our integrated network analysis suggested that the post-transcriptional downregulation of miRNAs and the corresponding upregulated expression of the target genes in the ovary of Finnsheep most likely account for the evolution of reproduction traits from the monotocous wild European mouflon to the highly prolific domestic Finnsheep (Fig. 3).

Regarding the 58 differentially expressed miRNAs, 210 predicted target genes and 1025 miRNA–gene pairs between Finnsheep and European mouflon, 27 miRNAs, 52 genes, and 67 miRNA–gene pairs have been reported in previous studies (Table S23), thus supporting our findings. Further studies involving a larger panel of species/breeds and sample sizes using qRT-PCR could help validate the relevance of the miRNAs, their target genes, and the miRNA–gene pairs revealed in this study.

## Conclusion

To our knowledge, this study provides the first mRNA–miRNA integrated profiling analysis of ovarian and endometrial tissues in wild and domestic sheep. By comparing the RNA-Seq data of the ovaries and endometria between European mouflon and Finnsheep, we identified differentially expressed mRNAs and miRNAs. The functional enrichment and miRNA–mRNA regulatory network analyses revealed novel genes and miRNAs associated with reproductive traits in wild and domestic sheep. Our results provide new insight into the molecular mechanisms and evolution of the high prolificacy traits in the *Ovis* species (Fig. 3). Additionally, the large amount of mRNA and miRNA data and the de novo transcriptome of European mouflon generated here provide valuable resources for studies investigating transcript functions associated with ovine reproduction traits under domestication and selection.

### Data archiving

The RNA-sequencing data reported in this article have been deposited in the Nucleotide and Sequence Read Archive databases (https://www.ncbi.nlm.nih.gov). The Illumina raw sequence reads appear in SRA under accession SRP142554 and under BioProject PRJNA451237.

## Electronic supplementary material

Supplementary Table S11

Supplementary Information

Supplementary Table S10
