# Supplementary material for: Comparative mRNA and miRNA expression in European mouflon (Ovis musimon) and sheep (Ovis aries) provides novel insights into the genetic mechanisms for female reproductive success
Source: Heredity (Edinb). 2018 May 21;122(2):172–86. doi: 10.1038/s41437-018-0090-1 (PMC6327046; doi:10.1038/s41437-018-0090-1)
Supplement: Supplementary file 2 — Supplementary Information [file 41437_2018_90_MOESM2_ESM.doc]

**Comparative mRNAs and miRNAs expression in European mouflon (*Ovis musimon*) and sheep (*Ovis aries*) provide novel insights into the genetic mechanisms for female reproductive success**

Ji Yang^1,†^, Xin Li^1,2,†^, Yin-Hong Cao^1,2,†^, Kisun Pokharel^3,4,†^, Xiao-Ju Hu^1,2,†^, Ze-Hui Chen^1,2,†^, Song-Song Xu^1,2^, Jaana Peippo^3^, Mervi Honkatukia^3^, Juha Kantanen^3^ & Meng-Hua Li^1,*^

**Supplementary Information includes：**

Supplementary Results

Supplementary Figs. S1–S5

Supplementary Tables S1–S23

Supplementary References

**Supplementary Results**

**Functional analysis of differentially expressed mRNAs between the ovarian and endometrial tissues**

In the four comparisons between ovary and endometrium tissues (M2-OA *vs*. M2-EA, M2-OA *vs*. M2-EB, M2-OB *vs*. M2-EA and M2-OB *vs*. M2-EB) in European mouflon, the functional GO enrichments of differentially expressed genes (DEGs) on the sheep reference genome (Oar v.4.0) were mainly associated with the developmental biological process (Table S16), whereas the enrichments on the *de novo* transcriptome were mostly involved in the metabolic process (Table S17). Despite distinct biological processes between the two approaches, the cellular component GO terms were both significantly enriched in membrane part, and the molecular function GO terms both in protein binding. Moreover, there are three significant enriched PANTHER pathways (*p*-value < 0.05) for the DEGs based on the *de novo* transcriptome (Table S18): integrin signaling pathway, CCKR signaling map pathway and TGF-beta signaling pathway. Among them, Integrin signaling pathway is the most significantly enriched one, in which integrins and adhesion molecules are up-regulated during different cellular processes such as adhesion, growth, proliferation, migration, survival and differentiation (Villegas-Pineda et al. 2015). In fact, the embryo implantation, a crucial process for the successful pregnancy, includes adhesion between trophoblast and endometrium. Also, the up-regulating expression of integrin heterodimer αVβ3 and αVβ5 can promote endometrial receptivity for embryo implantation (Chung et al. 2016). It was also suggested that integrins could provide directional signals that are necessary for transitioning of follicle stem cells (FSCs) from quiescence to proliferation in the *Drosophila* ovary (Hartman et al. 2015). These evidences suggested that Integrin signaling pathway may play an important role in ovary and endometrium of European mouflon. As for the other two significantly enriched pathways, CCKR in the CCKR signaling map pathway are [G protein-coupled receptors](http://topics.sciencedirect.com/topics/page/G_protein_coupled_receptors), which can elicit an [inositol trisphosphate](http://topics.sciencedirect.com/topics/page/Inositol_trisphosphate) (IP3)-induced calcium release from the [endoplasmic reticulum](http://topics.sciencedirect.com/topics/page/Endoplasmic_reticulum) (Staljanssens et al. 2012). The TGF-β signaling pathway has multiple functions such as follicle formation, ovulation and follicular development in the mammalian ovary. Also, it has been reported that blockade of TGF signaling could potentially increase infertility and pregnancy loss associated with uterine leiomyoma (Doherty and Taylor 2015). Thus, the two pathways appeared to be functionally relevant to ovary and endometrium as well.

**Complementary miRNA-gene regulatory network**

Besides those miRNAs in the ovarian main down-regulated network in Finnsheep (Fig. 4a), other miRNAs and their target genes identified in Finnsheep ovary (Table 2 and Fig. S4) could also have roles in regulating Finnsheep reproduction. In the Finnsheep ovarian down-regulated network (Fig. S4a), *miR-21* and *miR-143*, which targeted *INHBA* in the main network, are highly expressed in the ovary of sheep and other livestock like bovine (Hossain et al. 2012). *MiR-21* is an important regulator of the follicular-luteal transition in sheep (McBride et al. 2012), and is highly induced by luteinizing hormone (LH) in murine granulosa cells and related to the murine ovulation rate (Carletti et al. 2010). *MiR-143* controls human ovarian cell proliferation and apoptosis (Sirotkin et al. 2010), and inhibits the formation of murine primordial follicles by suppressing the proliferation of pregranulosa cells (Zhang et al. 2012). Similarly, *miR-340-5p* targeted the gene *SPP1* important for Finnsheep prolific trait as identified in the GO enrichment analysis of ovarian mRNAs (Table S14). *SPP1* has been also demonstrated to be directly associated with litter size in pig (Korwin-Kossakowska et al. 2002) and mapped to the region containing QTLs for porcine ovulation rate (Rathje et al. 1997) and litter size (King et al. 2003). *MiR-27a*, which targeted *KITLG* in the main network, is associated with porcine litter size (Lei et al. 2011). Moreover, in the Finnsheep ovarian up-regulated network (Fig. S4b), *let-7* miRNA family (e.g., *let-7c*, *let-7e-5p* in Table 2) is abundant in the livestock ovary (McBride et al. 2012), which have important regulatory roles in the process of follicular maturity (Murchison et al. 2007) and oocyte maturation (Pati and Habibi 2000; Song et al. 2014; Xiao et al. 2014). Collectively, the co-regulation of miRNAs and target genes discussed above may contribute to the prolificacy trait in Finnsheep as well.

For the miRNAs not included in the ovarian main down-regulated network for European mouflon (Fig. S4b), *miR-92a* is down-regulated during follicle atresia in human (Lin et al. 2012). *MiR-22-3p* is expressed at a low level in the premature ovarian failure (POF) and diminished ovarian reserve in Han Chinese (Dang et al. 2015). Also, in the European mouflon ovarian up-regulated network (Fig. S4a), *miR-10b*, which targeted *PIAS1* in the main network, is highly abundant in germinal vesicle (GV) oocytes in *Bos taurus* (Tripurani et al. 2010). *MiR-10b* is expressed at a high level in monotocous goat ovary and inhibits the proliferation of goat granulosa cells (Peng et al. 2016), and is up-regulated during follicle atresia in women (Lin et al. 2012). Furthermore, *miR-99a* is up-regulated during follicle atresia in porcine ovary (Lin et al. 2012), and *miR-27b* is predominately expressed in the ovaries of monotocous goats (An et al. 2016). These evidences indicated that the regulated expression of these miRNAs in European mouflon compared to Finnsheep may be closely related to the monotocous traits and the potential susceptibility to reproductive diseases in European mouflon.

In the GO enrichment analysis of the genes targeted by differentially expressed miRNAs between Finnsheep and European mouflon, the GO terms of the 100 up-regulated genes in European mouflon ovary (by 30 down-regulated miRNAs) were mainly associated with activation of protein kinase activity and positive regulation of transferase activity (Table S20). The 51 Finnsheep endometrium up-regulated target genes (by four down-regulated miRNAs) were significantly enriched in coenzyme biosynthetic process and response to organic substance (Table S21), whereas the 8 European mouflon endometrium up-regulated target genes (by one down-regulated miRNAs) were significantly enriched in tissue morphogenesis and heparin binding (Table S22).

Regarding the comparison of ovary and endometrium in European mouflon (Fig. S5), three endometrial up-regulated miRNAs (*miR-200b*, *miR-200a* and *miR-34c-5p*) targeted the gene *MAPK14*, while the ovarian up-regulated miRNA *miR-143* targeted *MAPK13*. The *MAPK13* and *MAPK14* genes both encode the MAPK family, which is functionally involved in the control of oocyte maturation in porcine (Sirotkin et al. 2010). As to the regulatory miRNAs, *miR-200b* has a central role in anovulation and infertility in female mice, and its inactivation could restrain LH biosynthesis and support ovulation by indirectly functioning in the hypothalamus-pituitary-ovarian axis (Hasuwa et al. 2013). Also, *miR-143* targets the gene *LIF*. *LIF/LIFR* system may serve a vital function in conceptus development and implantation in pigs (Geisert et al. 1998; Altmäe et al. 2013).

**Supplementary References**

[Altmäe S](https://www.ncbi.nlm.nih.gov/pubmed/?term=Altmäe S%5BAuthor%5D&cauthor=true&cauthor_uid=22902743), [Martinez-Conejero JA](https://www.ncbi.nlm.nih.gov/pubmed/?term=Martinez-Conejero JA%5BAuthor%5D&cauthor=true&cauthor_uid=22902743), [Esteban FJ](https://www.ncbi.nlm.nih.gov/pubmed/?term=Esteban FJ%5BAuthor%5D&cauthor=true&cauthor_uid=22902743), [Ruiz-Alonso M](https://www.ncbi.nlm.nih.gov/pubmed/?term=Ruiz-Alonso M%5BAuthor%5D&cauthor=true&cauthor_uid=22902743), [Stavreus-Evers A](https://www.ncbi.nlm.nih.gov/pubmed/?term=Stavreus-Evers A%5BAuthor%5D&cauthor=true&cauthor_uid=22902743), [Horcajadas JA](https://www.ncbi.nlm.nih.gov/pubmed/?term=Horcajadas JA%5BAuthor%5D&cauthor=true&cauthor_uid=22902743) et al. (2013). MicroRNAs *miR-30b*, *miR-30d*, and *miR-494* regulate human endometrial receptivity. *Reprod Sci* **20**:308**–**17.

An XP, Song YX, Hou JX, Zhang Y, Chen KW, Ma HD et al. (2016). Identification and profiling of microRNAs in the ovaries of polytocous and monotocous goats during estrus. *Theriogenology* **85**:769**–**80.

Carletti MZ, Fiedler SD, Christenson LK (2010). MicroRNA 21 blocks apoptosis in mouse periovulatory granulosa cells. *Biol Reprod* **83**:286**–**95.

# Chi SW, Zang JB, Mele A, Darnell RB (2009). Argonaute *HITS-CLIP* decodes microRNA–mRNA interaction maps. *Nature* 460:479–86.

Chung TW, Park MJ, Kim HS, Choi HJ, Ha KT (2016). Integrin *αVβ3* and *αVβ5* are required for leukemia inhibitory factor-mediated the adhesion of trophoblast cells to the endometrial cells. *Biochem Biophys Res Commun* **469**:936**–**40.

# Dai W, Wang C, Wang F, Wang Y, Shen M, Chen K et al. (2014). Anti-*miR-197* inhibits migration in *HCC* cells by targeting *KAI 1*/*CD82*. *Biochem Biophys Res Commun* 446:541–8.

Dang Y, Zhao S, Qin Y, Han T, Li W, Chen ZJ (2015). *MicroRNA-22-3p* is down-regulated in the plasma of Han Chinese patients with premature ovarian failure. *Fertil Steril* **103**:802**–**7.

Doherty LF, Taylor HS (2015). Leiomyoma-derived transforming growth factor-β impairs bone morphogenetic protein-2-mediated endometrial receptivity. *Fertil Steril* **103**:845**–**52.

# [Farazi TA](https://www.ncbi.nlm.nih.gov/pubmed/?term=Farazi TA%5BAuthor%5D&cauthor=true&cauthor_uid=24398324), [Ten Hoeve JJ](https://www.ncbi.nlm.nih.gov/pubmed/?term=Ten Hoeve JJ%5BAuthor%5D&cauthor=true&cauthor_uid=24398324), [Brown M](https://www.ncbi.nlm.nih.gov/pubmed/?term=Brown M%5BAuthor%5D&cauthor=true&cauthor_uid=24398324), [Mihailovic A](https://www.ncbi.nlm.nih.gov/pubmed/?term=Mihailovic A%5BAuthor%5D&cauthor=true&cauthor_uid=24398324), [Horlings HM](https://www.ncbi.nlm.nih.gov/pubmed/?term=Horlings HM%5BAuthor%5D&cauthor=true&cauthor_uid=24398324), [van de Vijver MJ](https://www.ncbi.nlm.nih.gov/pubmed/?term=van de Vijver MJ%5BAuthor%5D&cauthor=true&cauthor_uid=24398324), et al. (2014). Identification of distinct miRNA target regulation between breast cancer molecular subtypes using *AGO2-PAR-CLIP* and patient datasets. *Genome Biol* 15:R9.

# [Gabriely G](https://www.ncbi.nlm.nih.gov/pubmed/?term=Gabriely G%5BAuthor%5D&cauthor=true&cauthor_uid=18591254), [Wurdinger T](https://www.ncbi.nlm.nih.gov/pubmed/?term=Wurdinger T%5BAuthor%5D&cauthor=true&cauthor_uid=18591254), [Kesari S](https://www.ncbi.nlm.nih.gov/pubmed/?term=Kesari S%5BAuthor%5D&cauthor=true&cauthor_uid=18591254), [Esau CC](https://www.ncbi.nlm.nih.gov/pubmed/?term=Esau CC%5BAuthor%5D&cauthor=true&cauthor_uid=18591254), [Burchard J](https://www.ncbi.nlm.nih.gov/pubmed/?term=Burchard J%5BAuthor%5D&cauthor=true&cauthor_uid=18591254), [Linsley PS](https://www.ncbi.nlm.nih.gov/pubmed/?term=Linsley PS%5BAuthor%5D&cauthor=true&cauthor_uid=18591254) et al. (2008). MicroRNA 21 promotes glioma invasion by targeting matrix metalloproteinase regulators. *Mol Cell Biol* 28:5369–80.

Geisert RD, Yelich JV, Pratt T, Pomp D (1998). Expression of an inter-α-trypsin inhibitor heavy chain-like protein in the pig endometrium during the oestrous cycle and early pregnancy. *J Reprod Fertil* **114**:35**–**43.

# [Gennarino VA](https://www.ncbi.nlm.nih.gov/pubmed/?term=Gennarino VA%5BAuthor%5D&cauthor=true&cauthor_uid=19088304), [Sardiello M](https://www.ncbi.nlm.nih.gov/pubmed/?term=Sardiello M%5BAuthor%5D&cauthor=true&cauthor_uid=19088304), [Avellino R](https://www.ncbi.nlm.nih.gov/pubmed/?term=Avellino R%5BAuthor%5D&cauthor=true&cauthor_uid=19088304), [Meola N](https://www.ncbi.nlm.nih.gov/pubmed/?term=Meola N%5BAuthor%5D&cauthor=true&cauthor_uid=19088304), [Maselli V](https://www.ncbi.nlm.nih.gov/pubmed/?term=Maselli V%5BAuthor%5D&cauthor=true&cauthor_uid=19088304), [Anand S](https://www.ncbi.nlm.nih.gov/pubmed/?term=Anand S%5BAuthor%5D&cauthor=true&cauthor_uid=19088304) et al. (2009). MicroRNA target prediction by expression analysis of host genes. *Genome Res* 19:481–90.

# Grimson A, Farh KKH, Johnston WK, Garrett-Engele P, Lim LP, Bartel DP (2007). MicroRNA targeting specificity in mammals: determinants beyond seed pairing. *Mol Cell* 27:91–105.

# [Hafner M](https://www.ncbi.nlm.nih.gov/pubmed/?term=Hafner M%5BAuthor%5D&cauthor=true&cauthor_uid=20371350), [Landthaler M](https://www.ncbi.nlm.nih.gov/pubmed/?term=Landthaler M%5BAuthor%5D&cauthor=true&cauthor_uid=20371350), [Burger L](https://www.ncbi.nlm.nih.gov/pubmed/?term=Burger L%5BAuthor%5D&cauthor=true&cauthor_uid=20371350), [Khorshid M](https://www.ncbi.nlm.nih.gov/pubmed/?term=Khorshid M%5BAuthor%5D&cauthor=true&cauthor_uid=20371350), [Hausser J](https://www.ncbi.nlm.nih.gov/pubmed/?term=Hausser J%5BAuthor%5D&cauthor=true&cauthor_uid=20371350), [Berninger P](https://www.ncbi.nlm.nih.gov/pubmed/?term=Berninger P%5BAuthor%5D&cauthor=true&cauthor_uid=20371350) et al. (2010). Transcriptome-wide identification of RNA-binding protein and microRNA target sites by *PAR-CLIP.* *Cell* 141:129–41.

Hartman TR, Ventresca EM, Hopkins A, [Zinshteyn](https://www.ncbi.nlm.nih.gov/pubmed/?term=Zinshteyn D%5BAuthor%5D&cauthor=true&cauthor_uid=25680813) D, [Singh](https://www.ncbi.nlm.nih.gov/pubmed/?term=Singh T%5BAuthor%5D&cauthor=true&cauthor_uid=25680813) T, [O’Brien](https://www.ncbi.nlm.nih.gov/pubmed/?term=O%26%23x02019%3BBrien JA%5BAuthor%5D&cauthor=true&cauthor_uid=25680813) JA et al. (2015). Novel tools for genetic manipulation of follicle stem cells in the *Drosophila* ovary reveal an integrin-dependent transition from quiescence to proliferation. *Genetics* **199**:935**–**57.

Hasuwa H, Ueda J, Ikawa M, Okabe M (2013). *MiR-200b* and *miR-429* function in mouse ovulation and are essential for female fertility. *Science* **341**:71**–**3.

Helwak A, Kudla G, Dudnakova T, Tollervey D (2013). Mapping the human miRNA interactome by *CLASH* reveals frequent noncanonical binding. *Cell* **153**:654–65.

Hossain MM, Sohel MMH, Schellander K, Tesfaye D (2012). Characterization and importance of microRNAs in mammalian gonadal functions. *Cell Tissue Res* **349**:679**–**90.

[Hu X](https://www.ncbi.nlm.nih.gov/pubmed/?term=Hu X%5BAuthor%5D&cauthor=true&cauthor_uid=26582387)J, [Pokharel K](https://www.ncbi.nlm.nih.gov/pubmed/?term=Pokharel K%5BAuthor%5D&cauthor=true&cauthor_uid=26582387), [Peippo J](https://www.ncbi.nlm.nih.gov/pubmed/?term=Peippo J%5BAuthor%5D&cauthor=true&cauthor_uid=26582387), [Ghanem N](https://www.ncbi.nlm.nih.gov/pubmed/?term=Ghanem N%5BAuthor%5D&cauthor=true&cauthor_uid=26582387), [Zhaboyev I](https://www.ncbi.nlm.nih.gov/pubmed/?term=Zhaboyev I%5BAuthor%5D&cauthor=true&cauthor_uid=26582387), [Kantanen J](https://www.ncbi.nlm.nih.gov/pubmed/?term=Kantanen J%5BAuthor%5D&cauthor=true&cauthor_uid=26582387) et al. (2016). Identification and characterization of miRNAs in the ovaries of a highly prolific sheep breed. *Anim Genet* **47**:234–9.

# Karginov FV, Hannon GJ (2013). Remodeling of *Ago2*-mRNA interactions upon cellular stress reflects miRNA complementarity and correlates with altered translation rates. *Genes Dev* 27:1624–32.

King AH, Jiang Z, Gibson JP, Haley CS, Archibald AL (2003). Mapping quantitative trait loci affecting female reproductive traits on porcine chromosome 8. *Biol Reprod* **68**:2172**–**9.

# Kishore S, Jaskiewicz L, Burger L, Hausser J, Khorshid M, Zavolan M (2011). A quantitative analysis of *CLIP* methods for identifying binding sites of RNA-binding proteins. *Nat Methods* 8:559–64.

Korwin-Kossakowska A, Kamyczek M, Cieslak D, Pierzchala M, Kuryl J (2002). The effect of the polymorphism of leptin (*LEP*), leptin receptor (*LEPR*) and osteopontin (*OPN*) genes on selected reproduction traits of synthetic Line 990 sows. *Anim Sci Pap Rep* **20**:159**–**68.

[Lei B](https://www.ncbi.nlm.nih.gov/pubmed/?term=Lei B%5BAuthor%5D&cauthor=true&cauthor_uid=21104015), [Gao S](https://www.ncbi.nlm.nih.gov/pubmed/?term=Gao S%5BAuthor%5D&cauthor=true&cauthor_uid=21104015), [Luo LF](https://www.ncbi.nlm.nih.gov/pubmed/?term=Luo LF%5BAuthor%5D&cauthor=true&cauthor_uid=21104015), [Xia XY](https://www.ncbi.nlm.nih.gov/pubmed/?term=Xia XY%5BAuthor%5D&cauthor=true&cauthor_uid=21104015), [Jiang SW](https://www.ncbi.nlm.nih.gov/pubmed/?term=Jiang SW%5BAuthor%5D&cauthor=true&cauthor_uid=21104015), [Deng CY](https://www.ncbi.nlm.nih.gov/pubmed/?term=Deng CY%5BAuthor%5D&cauthor=true&cauthor_uid=21104015) et al. (2011). A SNP in the *miR-27a* gene is associated with litter size in pigs. *Mol Biol Rep* **38**:3725**–**9.

# [Leung AK](https://www.ncbi.nlm.nih.gov/pubmed/?term=Leung AK%5BAuthor%5D&cauthor=true&cauthor_uid=21258322), [Young AG](https://www.ncbi.nlm.nih.gov/pubmed/?term=Young AG%5BAuthor%5D&cauthor=true&cauthor_uid=21258322), [Bhutkar A](https://www.ncbi.nlm.nih.gov/pubmed/?term=Bhutkar A%5BAuthor%5D&cauthor=true&cauthor_uid=21258322), [Zheng GX](https://www.ncbi.nlm.nih.gov/pubmed/?term=Zheng GX%5BAuthor%5D&cauthor=true&cauthor_uid=21258322), [Bosson AD](https://www.ncbi.nlm.nih.gov/pubmed/?term=Bosson AD%5BAuthor%5D&cauthor=true&cauthor_uid=21258322), [Nielsen CB](https://www.ncbi.nlm.nih.gov/pubmed/?term=Nielsen CB%5BAuthor%5D&cauthor=true&cauthor_uid=21258322) et al. (2011). Genome-wide identification of *Ago2* binding sites from mouse embryonic stem cells with and without mature microRNAs. *Nat Struct Mol Biol* 18:237–44.

Lin F, Li R, Pan ZX, Zhou B, Yu DB, Wang XG et al. (2012). *MiR-26b* promotes granulosa cell apoptosis by targeting *ATM* during follicular atresia in porcine ovary. *PLoS One* **7**:e38640.

# [Lipchina I](https://www.ncbi.nlm.nih.gov/pubmed/?term=Lipchina I%5BAuthor%5D&cauthor=true&cauthor_uid=22012620), [Elkabetz Y](https://www.ncbi.nlm.nih.gov/pubmed/?term=Elkabetz Y%5BAuthor%5D&cauthor=true&cauthor_uid=22012620), [Hafner M](https://www.ncbi.nlm.nih.gov/pubmed/?term=Hafner M%5BAuthor%5D&cauthor=true&cauthor_uid=22012620), [Sheridan R](https://www.ncbi.nlm.nih.gov/pubmed/?term=Sheridan R%5BAuthor%5D&cauthor=true&cauthor_uid=22012620), [Mihailovic A](https://www.ncbi.nlm.nih.gov/pubmed/?term=Mihailovic A%5BAuthor%5D&cauthor=true&cauthor_uid=22012620), [Tuschl T](https://www.ncbi.nlm.nih.gov/pubmed/?term=Tuschl T%5BAuthor%5D&cauthor=true&cauthor_uid=22012620) et al. (2011). Genome-wide identification of microRNA targets in human *ES* cells reveals a role for *miR-302* in modulating *BMP* response. *Genes Dev* 25:2173–86.

# [Loeb GB](https://www.ncbi.nlm.nih.gov/pubmed/?term=Loeb GB%5BAuthor%5D&cauthor=true&cauthor_uid=23142080), [Khan AA](https://www.ncbi.nlm.nih.gov/pubmed/?term=Khan AA%5BAuthor%5D&cauthor=true&cauthor_uid=23142080), [Canner D](https://www.ncbi.nlm.nih.gov/pubmed/?term=Canner D%5BAuthor%5D&cauthor=true&cauthor_uid=23142080), [Hiatt JB](https://www.ncbi.nlm.nih.gov/pubmed/?term=Hiatt JB%5BAuthor%5D&cauthor=true&cauthor_uid=23142080), [Shendure J](https://www.ncbi.nlm.nih.gov/pubmed/?term=Shendure J%5BAuthor%5D&cauthor=true&cauthor_uid=23142080), [Darnell RB](https://www.ncbi.nlm.nih.gov/pubmed/?term=Darnell RB%5BAuthor%5D&cauthor=true&cauthor_uid=23142080) et al. (2012). Transcriptome-wide *miR-155* binding map reveals widespread noncanonical microRNA targeting. *Mol Cell* 48:760–70.

[McBride D](https://www.ncbi.nlm.nih.gov/pubmed/?term=McBride D%5BAuthor%5D&cauthor=true&cauthor_uid=22653318), [Carré W](https://www.ncbi.nlm.nih.gov/pubmed/?term=Carré W%5BAuthor%5D&cauthor=true&cauthor_uid=22653318), [Sontakke SD](https://www.ncbi.nlm.nih.gov/pubmed/?term=Sontakke SD%5BAuthor%5D&cauthor=true&cauthor_uid=22653318), [Hogg CO](https://www.ncbi.nlm.nih.gov/pubmed/?term=Hogg CO%5BAuthor%5D&cauthor=true&cauthor_uid=22653318), [Law A](https://www.ncbi.nlm.nih.gov/pubmed/?term=Law A%5BAuthor%5D&cauthor=true&cauthor_uid=22653318), [Donadeu FX](https://www.ncbi.nlm.nih.gov/pubmed/?term=Donadeu FX%5BAuthor%5D&cauthor=true&cauthor_uid=22653318) et al. (2012). Identification of miRNAs associated with the follicular–luteal transition in the ruminant ovary. *Reproduction* **144**:221**–**33.

# Melton C, Judson RL, Blelloch R (2010). Opposing microRNA families regulate self-renewal in mouse embryonic stem cells. *Nature* 463:621–6.

# [Memczak S](https://www.ncbi.nlm.nih.gov/pubmed/?term=Memczak S%5BAuthor%5D&cauthor=true&cauthor_uid=23446348), [Jens M](https://www.ncbi.nlm.nih.gov/pubmed/?term=Jens M%5BAuthor%5D&cauthor=true&cauthor_uid=23446348), [Elefsinioti A](https://www.ncbi.nlm.nih.gov/pubmed/?term=Elefsinioti A%5BAuthor%5D&cauthor=true&cauthor_uid=23446348), [Torti F](https://www.ncbi.nlm.nih.gov/pubmed/?term=Torti F%5BAuthor%5D&cauthor=true&cauthor_uid=23446348), [Krueger J](https://www.ncbi.nlm.nih.gov/pubmed/?term=Krueger J%5BAuthor%5D&cauthor=true&cauthor_uid=23446348), [Rybak A](https://www.ncbi.nlm.nih.gov/pubmed/?term=Rybak A%5BAuthor%5D&cauthor=true&cauthor_uid=23446348) et al. (2013). Circular RNAs are a large class of animal RNAs with regulatory potency. *Nature* 495:333–8.

[Murchison EP](https://www.ncbi.nlm.nih.gov/pubmed/?term=Murchison EP%5BAuthor%5D&cauthor=true&cauthor_uid=17369401), [Stein P](https://www.ncbi.nlm.nih.gov/pubmed/?term=Stein P%5BAuthor%5D&cauthor=true&cauthor_uid=17369401), [Xuan Z](https://www.ncbi.nlm.nih.gov/pubmed/?term=Xuan Z%5BAuthor%5D&cauthor=true&cauthor_uid=17369401), [Pan H](https://www.ncbi.nlm.nih.gov/pubmed/?term=Pan H%5BAuthor%5D&cauthor=true&cauthor_uid=17369401), [Zhang MQ](https://www.ncbi.nlm.nih.gov/pubmed/?term=Zhang MQ%5BAuthor%5D&cauthor=true&cauthor_uid=17369401), [Schultz RM](https://www.ncbi.nlm.nih.gov/pubmed/?term=Schultz RM%5BAuthor%5D&cauthor=true&cauthor_uid=17369401) et al. (2007). Critical roles for Dicer in the female germline. *Genes Dev* **21**:682**–**93.

Pati D, Habibi HR (2000). Direct action of *GnRH* variants on goldfish oocyte meiosis and follicular steroidogenesis. *Mol Cell Endocrinol* **160**:75**–**88.

[Peng JY](https://www.ncbi.nlm.nih.gov/pubmed/?term=Peng JY%5BAuthor%5D&cauthor=true&cauthor_uid=26513157), [An XP](https://www.ncbi.nlm.nih.gov/pubmed/?term=An XP%5BAuthor%5D&cauthor=true&cauthor_uid=26513157), [Fang F](https://www.ncbi.nlm.nih.gov/pubmed/?term=Fang F%5BAuthor%5D&cauthor=true&cauthor_uid=26513157), [Gao KX](https://www.ncbi.nlm.nih.gov/pubmed/?term=Gao KX%5BAuthor%5D&cauthor=true&cauthor_uid=26513157), [Xin HY](https://www.ncbi.nlm.nih.gov/pubmed/?term=Xin HY%5BAuthor%5D&cauthor=true&cauthor_uid=26513157), [Han P](https://www.ncbi.nlm.nih.gov/pubmed/?term=Han P%5BAuthor%5D&cauthor=true&cauthor_uid=26513157) et al. (2016). *MicroRNA-10b* suppresses goat granulosa cell proliferation by targeting brain-derived neurotropic factor. *Domest Anim Endocrinol* **54**:60**–**7.

Rathje TA, Rohrer GA, Johnson RK (1997). Evidence for quantitative trait loci affecting ovulation rate in pigs. *J Anim Sci* **75**:1486**–**94.

# Riley KJ, Rabinowitz GS, Yario TA, Luna JM, Darnell RB, Steitz JA (2012). *EBV* and human microRNAs co-target oncogenic and apoptotic viral and human genes during latency. *EMBO J* 31:2207–21.

Sirotkin AV, Lauková M, Ovcharenko D, Brenaut P, Mlynček M (2010). Identification of microRNAs controlling human ovarian cell proliferation and apoptosis. *J Cell Physio* **223**:49**–**56.

# [Skalsky RL](https://www.ncbi.nlm.nih.gov/pubmed/?term=Skalsky RL%5BAuthor%5D&cauthor=true&cauthor_uid=22291592), [Corcoran DL](https://www.ncbi.nlm.nih.gov/pubmed/?term=Corcoran DL%5BAuthor%5D&cauthor=true&cauthor_uid=22291592), [Gottwein E](https://www.ncbi.nlm.nih.gov/pubmed/?term=Gottwein E%5BAuthor%5D&cauthor=true&cauthor_uid=22291592), [Frank CL](https://www.ncbi.nlm.nih.gov/pubmed/?term=Frank CL%5BAuthor%5D&cauthor=true&cauthor_uid=22291592), [Kang D](https://www.ncbi.nlm.nih.gov/pubmed/?term=Kang D%5BAuthor%5D&cauthor=true&cauthor_uid=22291592), [Hafner M](https://www.ncbi.nlm.nih.gov/pubmed/?term=Hafner M%5BAuthor%5D&cauthor=true&cauthor_uid=22291592) et al. (2012). The viral and cellular microRNA targetome in lymphoblastoid cell lines. *PLoS Pathog* 8:e1002484.

Song YN, Shi LL, Liu ZQ, Qiu GF (2014). Global analysis of the ovarian microRNA transcriptome: implication for *miR-2* and *miR-133* regulation of oocyte meiosis in the Chinese mitten crab, *Eriocheir sinensis* (Crustacea: Decapoda). *BMC Genomics* **15**:547.

Staljanssens D, De Vos WH, Willems P, Van Camp J, Smagghe G (2012). Time-resolved quantitative analysis of *CCK1* receptor-induced intracellular calcium increase. *Peptides* **34**:219**–**25.

# [Tavazoie SF](https://www.ncbi.nlm.nih.gov/pubmed/?term=Tavazoie SF%5BAuthor%5D&cauthor=true&cauthor_uid=18185580), [Alarcón C](https://www.ncbi.nlm.nih.gov/pubmed/?term=Alarcón C%5BAuthor%5D&cauthor=true&cauthor_uid=18185580), [Oskarsson T](https://www.ncbi.nlm.nih.gov/pubmed/?term=Oskarsson T%5BAuthor%5D&cauthor=true&cauthor_uid=18185580), [Padua D](https://www.ncbi.nlm.nih.gov/pubmed/?term=Padua D%5BAuthor%5D&cauthor=true&cauthor_uid=18185580), [Wang Q](https://www.ncbi.nlm.nih.gov/pubmed/?term=Wang Q%5BAuthor%5D&cauthor=true&cauthor_uid=18185580), [Bos PD](https://www.ncbi.nlm.nih.gov/pubmed/?term=Bos PD%5BAuthor%5D&cauthor=true&cauthor_uid=18185580) et al. (2008). Endogenous human microRNAs that suppress breast cancer metastasis. *Nature* 451:147–52.

Tripurani SK, Xiao C, Salem M, Yao J (2010). Cloning and analysis of fetal ovary microRNAs in cattle. *Anim Reprod Sci* **120**:16**–**22.

# Tseng CW, Lin CC, Chen CN, Huang HC, Juan HF (2011). Integrative network analysis reveals active microRNAs and their functions in gastric cancer. *BMC Syst Biol* 5:99.

# [Villadsen SB](https://www.ncbi.nlm.nih.gov/pubmed/?term=Villadsen SB%5BAuthor%5D&cauthor=true&cauthor_uid=22108519), [Bramsen JB](https://www.ncbi.nlm.nih.gov/pubmed/?term=Bramsen JB%5BAuthor%5D&cauthor=true&cauthor_uid=22108519), [Ostenfeld MS](https://www.ncbi.nlm.nih.gov/pubmed/?term=Ostenfeld MS%5BAuthor%5D&cauthor=true&cauthor_uid=22108519), [Wiklund ED](https://www.ncbi.nlm.nih.gov/pubmed/?term=Wiklund ED%5BAuthor%5D&cauthor=true&cauthor_uid=22108519), [Fristrup N](https://www.ncbi.nlm.nih.gov/pubmed/?term=Fristrup N%5BAuthor%5D&cauthor=true&cauthor_uid=22108519), [Gao S](https://www.ncbi.nlm.nih.gov/pubmed/?term=Gao S%5BAuthor%5D&cauthor=true&cauthor_uid=22108519) et al. (2012). The *miR-143*/*-145* cluster regulates plasminogen activator inhibitor-1 in bladder cancer. *Br J Cancer* 106:366–74.

Villegas-Pineda JC, Garibay-Cerdenares OL, Hernández-Ramírez VI, Gallardo-Rincón D, de León DC, Pérez-Montiel-Gómez MD et al. (2015). Integrins and haptoglobin: Molecules overexpressed in ovarian cancer. *Pathol Res Pract* **211**:973**–**81.

# [Whisnant AW](https://www.ncbi.nlm.nih.gov/pubmed/?term=Whisnant AW%5BAuthor%5D&cauthor=true&cauthor_uid=23592263), [Bogerd HP](https://www.ncbi.nlm.nih.gov/pubmed/?term=Bogerd HP%5BAuthor%5D&cauthor=true&cauthor_uid=23592263), [Flores O](https://www.ncbi.nlm.nih.gov/pubmed/?term=Flores O%5BAuthor%5D&cauthor=true&cauthor_uid=23592263), [Ho P](https://www.ncbi.nlm.nih.gov/pubmed/?term=Ho P%5BAuthor%5D&cauthor=true&cauthor_uid=23592263), [Powers JG](https://www.ncbi.nlm.nih.gov/pubmed/?term=Powers JG%5BAuthor%5D&cauthor=true&cauthor_uid=23592263), [Sharova N](https://www.ncbi.nlm.nih.gov/pubmed/?term=Sharova N%5BAuthor%5D&cauthor=true&cauthor_uid=23592263) et al. (2013). In-depth analysis of the interaction of *HIV-1* with cellular microRNA biogenesis and effector mechanisms. *Mbio* 4:e000193.

# White NMA, Masui O, Newsted D, Scorilas A, Romaschin AD, Bjarnason GA et al. (2014). Galectin-1 has potential prognostic significance and is implicated in clear cell renal cell carcinoma progression through the HIF/mTOR signaling axis. *Br J Cancer* 110:1250–9.

[Xiao G](https://www.ncbi.nlm.nih.gov/pubmed/?term=Xiao G%5BAuthor%5D&cauthor=true&cauthor_uid=24959893), [Xia C](https://www.ncbi.nlm.nih.gov/pubmed/?term=Xia C%5BAuthor%5D&cauthor=true&cauthor_uid=24959893), [Yang J](https://www.ncbi.nlm.nih.gov/pubmed/?term=Yang J%5BAuthor%5D&cauthor=true&cauthor_uid=24959893), [Liu J](https://www.ncbi.nlm.nih.gov/pubmed/?term=Liu J%5BAuthor%5D&cauthor=true&cauthor_uid=24959893), [Du H](https://www.ncbi.nlm.nih.gov/pubmed/?term=Du H%5BAuthor%5D&cauthor=true&cauthor_uid=24959893), [Kang X](https://www.ncbi.nlm.nih.gov/pubmed/?term=Kang X%5BAuthor%5D&cauthor=true&cauthor_uid=24959893) et al. (2014). *MiR-133b* regulates the expression of the Actin protein *TAGLN2* during oocyte growth and maturation: a potential target for infertility therapy. *PLoS One* **9**:e100751.

# Xue Y, Ouyang K, Huang J, [Zhou Y](https://www.ncbi.nlm.nih.gov/pubmed/?term=Zhou Y%5BAuthor%5D&cauthor=true&cauthor_uid=23313552), [Ouyang H](https://www.ncbi.nlm.nih.gov/pubmed/?term=Ouyang H%5BAuthor%5D&cauthor=true&cauthor_uid=23313552), [Li H](https://www.ncbi.nlm.nih.gov/pubmed/?term=Li H%5BAuthor%5D&cauthor=true&cauthor_uid=23313552) et al. (2013). Direct conversion of fibroblasts to neurons by reprogramming *PTB*-regulated microRNA circuits. *Cell* 152:82–96.

[Zhang J](https://www.ncbi.nlm.nih.gov/pubmed/?term=Zhang J%5BAuthor%5D&cauthor=true&cauthor_uid=23276944), [Ji X](https://www.ncbi.nlm.nih.gov/pubmed/?term=Ji X%5BAuthor%5D&cauthor=true&cauthor_uid=23276944), [Zhou D](https://www.ncbi.nlm.nih.gov/pubmed/?term=Zhou D%5BAuthor%5D&cauthor=true&cauthor_uid=23276944), [Li Y](https://www.ncbi.nlm.nih.gov/pubmed/?term=Li Y%5BAuthor%5D&cauthor=true&cauthor_uid=23276944), [Lin J](https://www.ncbi.nlm.nih.gov/pubmed/?term=Lin J%5BAuthor%5D&cauthor=true&cauthor_uid=23276944), [Liu J](https://www.ncbi.nlm.nih.gov/pubmed/?term=Liu J%5BAuthor%5D&cauthor=true&cauthor_uid=23276944) et al. (2012). *MiR-143* is critical for the formation of primordial follicles in mice. *Front Biosci (Landmark Ed.)* **18**:588**–**97.

# [Zhang X](https://www.ncbi.nlm.nih.gov/pubmed/?term=Zhang X%5BAuthor%5D&cauthor=true&cauthor_uid=25083871), [Zuo X](https://www.ncbi.nlm.nih.gov/pubmed/?term=Zuo X%5BAuthor%5D&cauthor=true&cauthor_uid=25083871), [Yang B](https://www.ncbi.nlm.nih.gov/pubmed/?term=Yang B%5BAuthor%5D&cauthor=true&cauthor_uid=25083871) , [Li Z](https://www.ncbi.nlm.nih.gov/pubmed/?term=Li Z%5BAuthor%5D&cauthor=true&cauthor_uid=25083871) , [Xue Y](https://www.ncbi.nlm.nih.gov/pubmed/?term=Xue Y%5BAuthor%5D&cauthor=true&cauthor_uid=25083871), [Zhou Y](https://www.ncbi.nlm.nih.gov/pubmed/?term=Zhou Y%5BAuthor%5D&cauthor=true&cauthor_uid=25083871) et al. (2014). MicroRNA directly enhances mitochondrial translation during muscle differentiation. *Cell* 158:607–19.

# Zhu ED, Li N, Li BS, Li W, Zhang WJ, MAO XH et al. (2014). *MiR-30b*, down-regulated in gastric cancer, promotes apoptosis and suppresses tumor growth by targeting plasminogen activator inhibitor-1. *PLoS One* 9:e106049.

**Supplementary Figures**


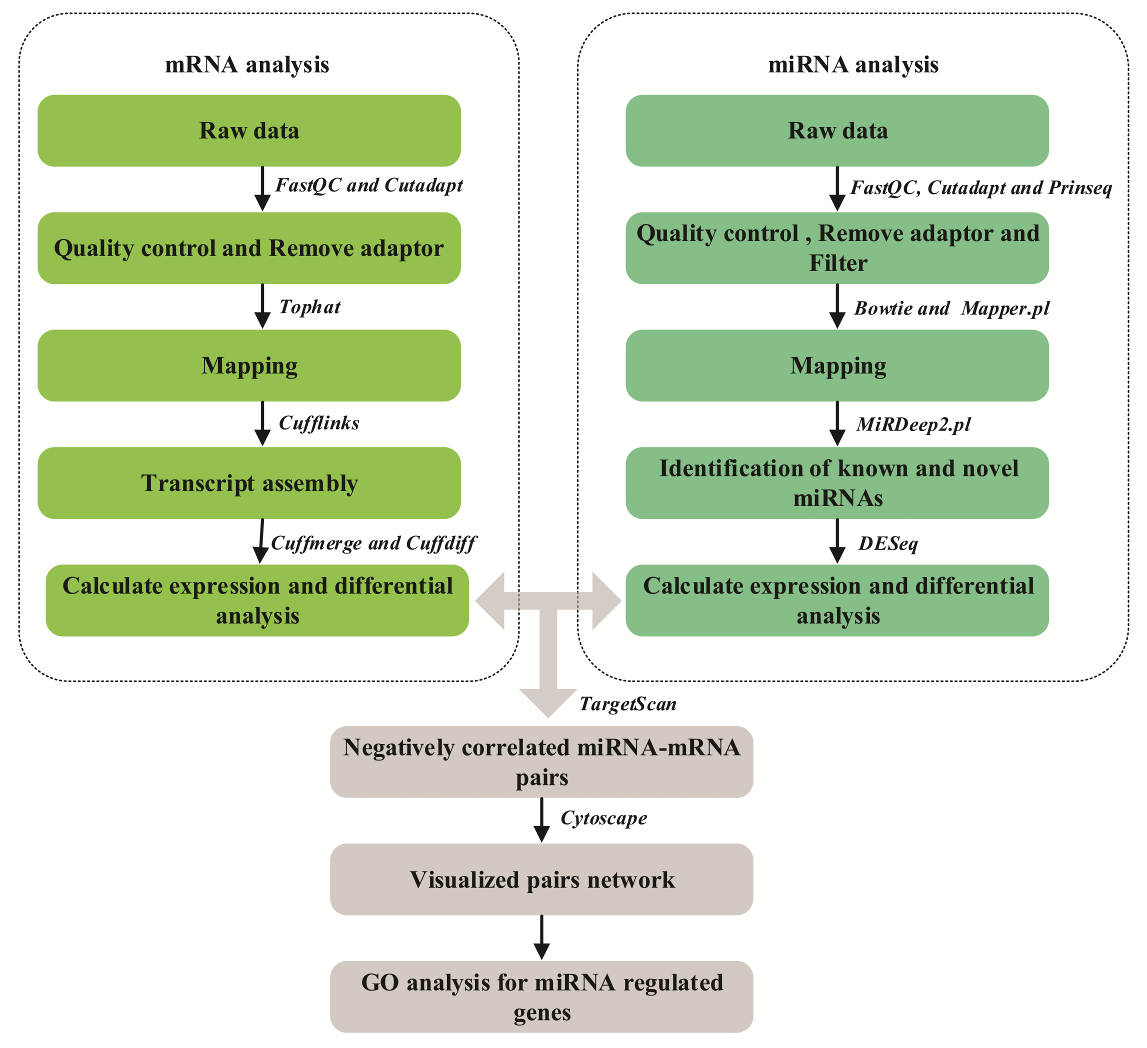


**Fig. S1.** The pipeline of mRNA analysis, miRNA analysis and miRNA-mRNA integrated analysis. Software and tools are shown in italics.


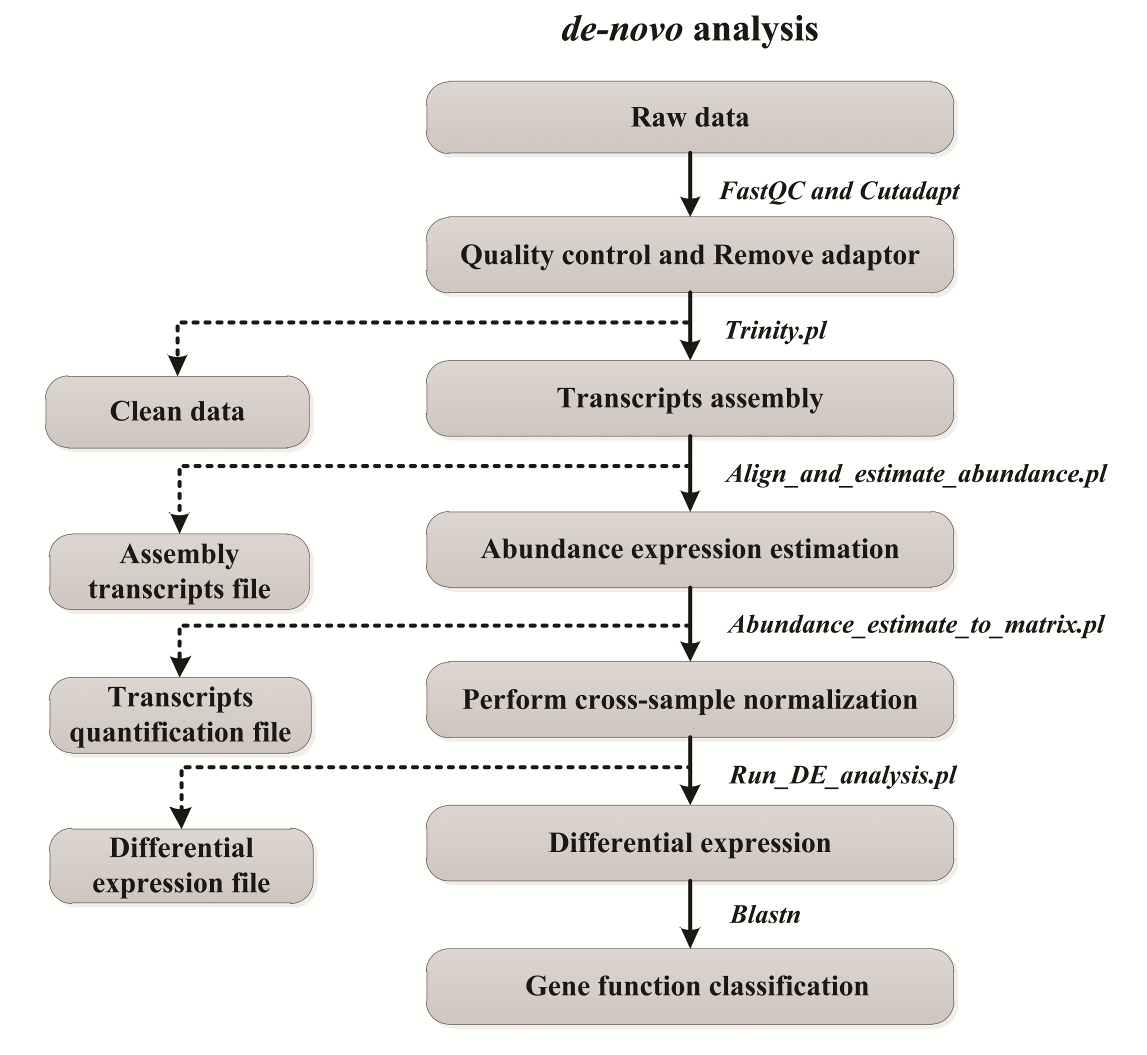


**Fig. S2.** The pipeline of de novo assembly and differential expression analyses on European mouflon ovarian and endometrial mRNA sequences. Software and tools are shown in italics.

**
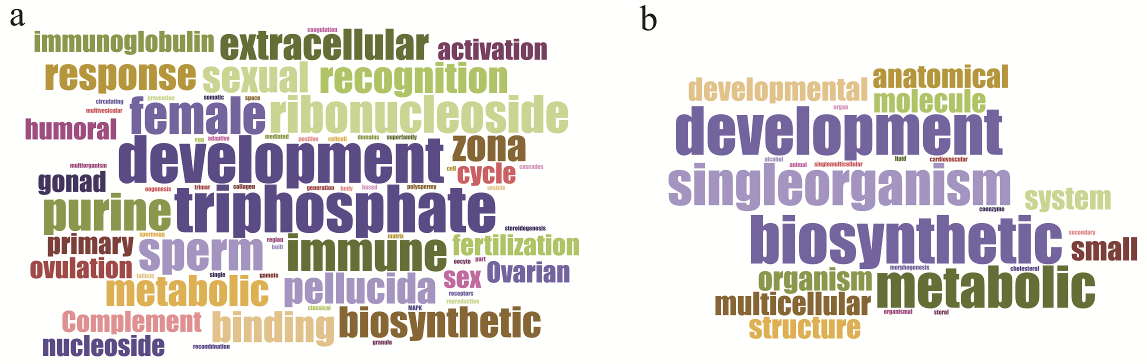
**

**Fig. S3.** Word cloud illustrating major enrichment in gene ontology terms and pathways for differentially expressed genes in the tissues of ovary **a** and up-regulated genes in the tissues of endometrium **b** between European mouflon and Finnsheep.


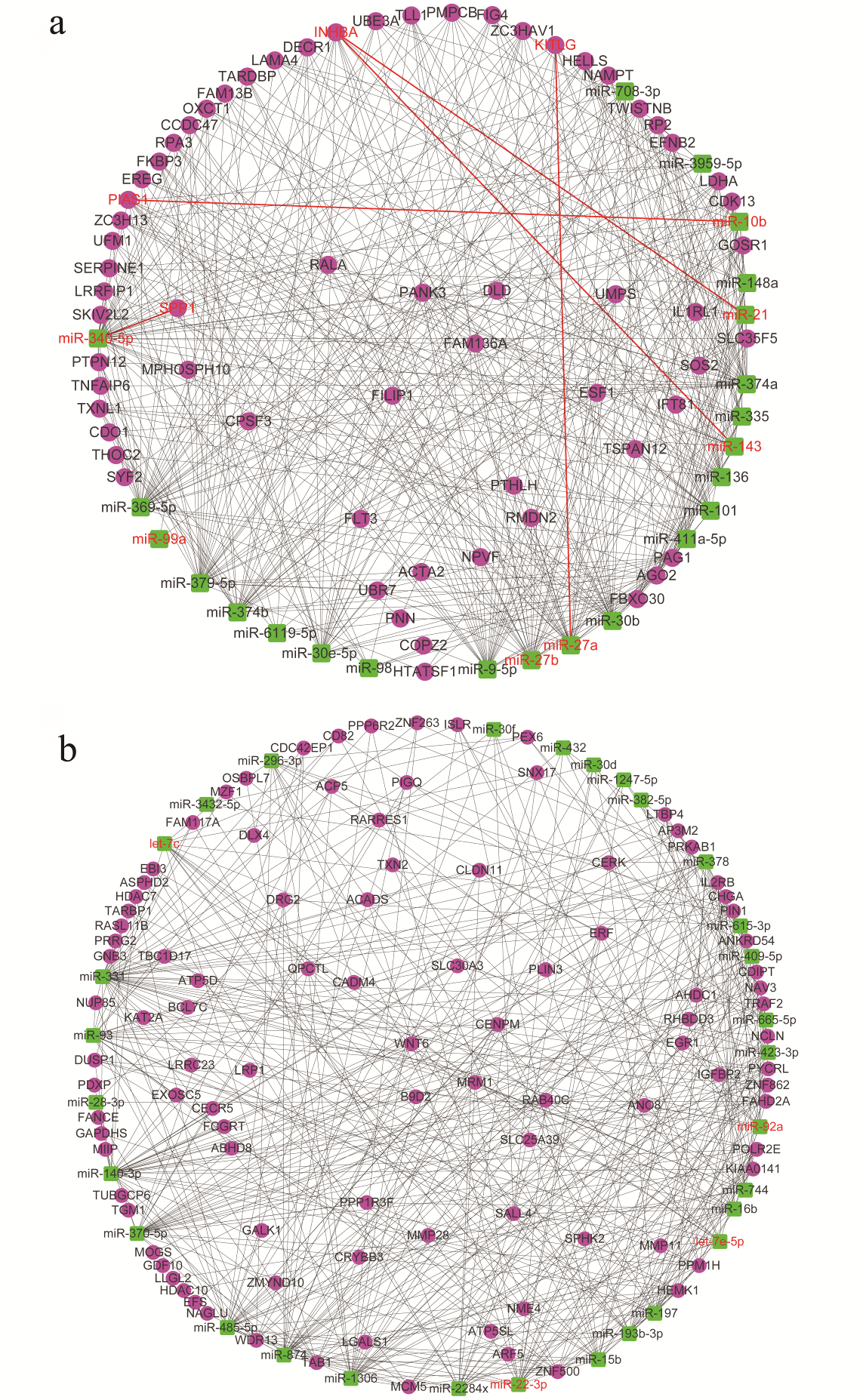


**Fig. S4.** The miRNA-mRNA regulatory network for sheep ovary. (A) The ovarian network of Finnsheep down-regulated (i.e., European mouflon up-regulated) miRNAs and target genes. (B) The ovarian network of European mouflon down-regulated (i.e., Finnsheep up-regulated) miRNAs and target genes. The round-rectangle and ellipse represent miRNAs and target genes, and the purple and green colors indicate up-regulation and down-regulation, respectively. The important miRNAs, genes and miRNA-gene pairs discussed in the Supplementary results are marked with red color.


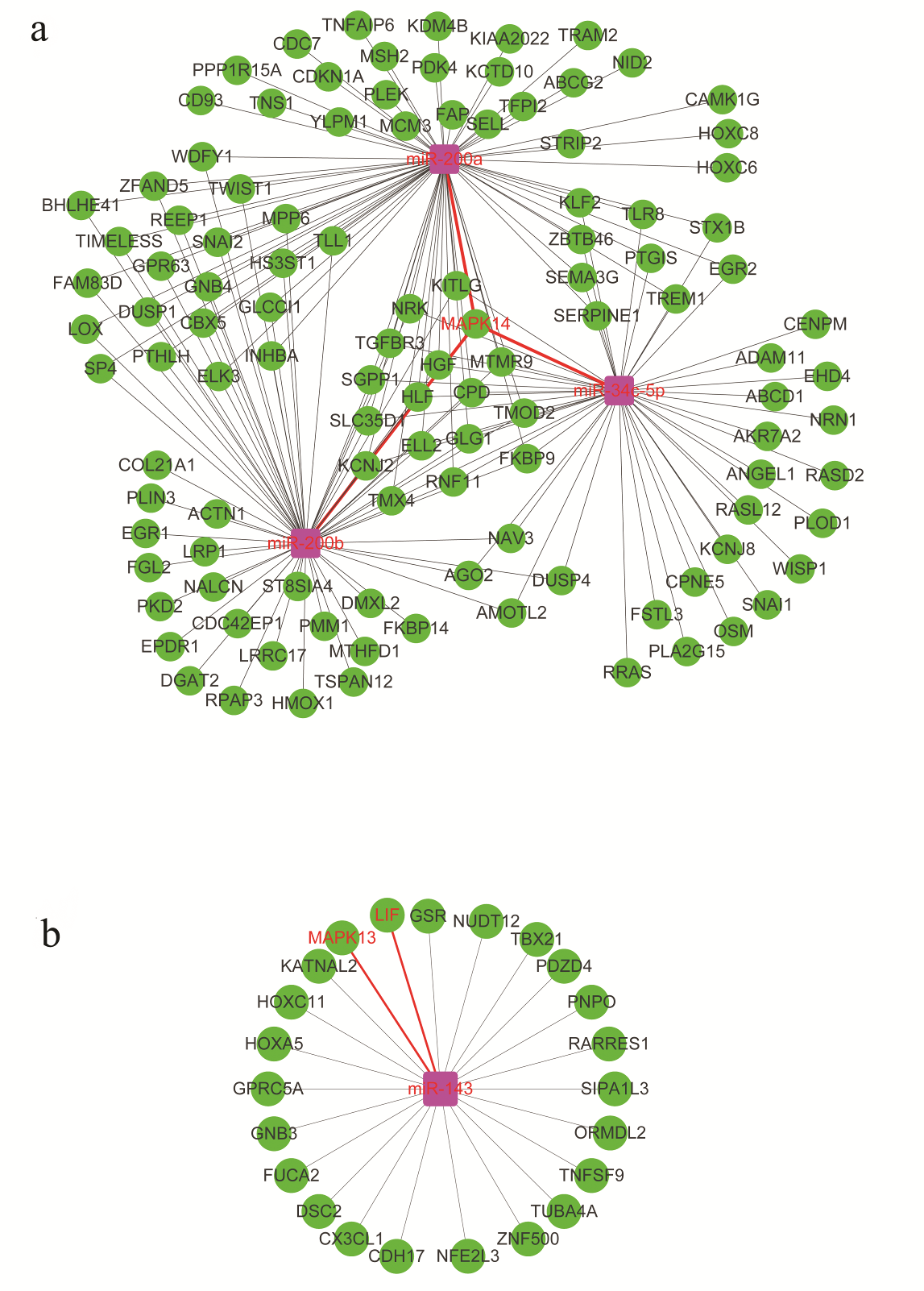


**Fig. S5.** The miRNA-mRNA regulatory network for the comparison of ovary and endometrium in European mouflon. (A) The endometrial up-regulated (i.e., ovarian down-regulated) miRNAs and target genes. (B) The ovarian up-regulated (i.e., endometrial down-regulated) miRNAs and target genes. The round-rectangle and ellipse represent miRNAs and target genes, and the purple and green colors indicate up-regulation and down-regulation, respectively. The important miRNAs, genes and miRNA-gene pairs discussed in the Supplementary Results are marked with red color.

**Supplementary Tables**

**Table S1.** Samples analyzed for mRNA and miRNA profiling in European mouflon and Finnsheep

|  | Ovary | Endometrium |
| --- | --- | --- |
| European mouflon | 6 samples (M1-OA, M1-OB, M2-OA, M2-OB, M3-OA and M3-OB) | 2 samples (M2-EA and M2-EB) for mRNA analysis  1 sample (M2-EB) for miRNA analysis |
| Finnsheep | 6 samples (F1-O, F2-O, F3-O, F4-O, F5-O and F6-O) | 2 samples (F2-E and F3-E) |

**Table S2.** Summary information of the miRNA and mRNA sequencing reads

|  | | Ovary | | | Endometrium | | |
| --- | --- | --- | --- | --- | --- | --- | --- |
|  |  | European mouflon | Finnsheep | Total | European mouflon | Finnsheep | Total |
| miRNA | Raw reads | 48798730 | 121432203 | 170230933 | 11450615 | 12510788 | 23961403 |
|  | Clean reads | 45706039 | 116322953 | 162028992 | 11071731 | 11508595 | 22580326 |
|  | 18-26 nt^a^ reads | 33638999 | 69803118 | 103442117 | 6616008 | 6438283 | 13054291 |
|  | Mapped reads | 31622471 | 64436304 | 96058775 | 5943365 | 5531321 | 11474686 |
| mRNA | Raw reads | 883816080 | 726675310 | 1610491390 | 200060940 | 207941256 | 408002196 |
|  | Clean reads | 883539837 | 726675310 | 1610215417 | 200060940 | 207941256 | 408002196 |
|  | Mapped reads | 882798001 | 723763080 | 1606561081 | 200030833 | 207896856 | 407927689 |

^a^18-26 nt denotes the miRNA sequences in 18-26 nucleotides.

**Table S3.** Summary information of the gene expression level based on the aligned mRNA reads to the Oar v.4.0 genome as measured by the FPKM (fragments per kilobase of exon per million fragments mapped) values

| FPKM | Ovary | | Endometrium | |
| --- | --- | --- | --- | --- |
|  | European mouflon | Finnsheep | European mouflon | Finnsheep |
| 0-50 | 22403 (93.03%) | 24830 (93.47%) | 18935 (92.02%) | 18170 (89.23%) |
| 50-100 | 874 (3.63%) | 924 (3.48%) | 899 (4.37%) | 1119 (5.50%) |
| 100-500 | 640 (2.66%) | 647 (2.44%) | 581 (2.82%) | 854 (4.19%) |
| >500 | 164 (0.68%) | 164 (0.62%) | 162 (0.79%) | 219 (1.08%) |
| total | 24081 | 26565 | 20577 | 20362 |

**Table S4.** The number of miRNAs identified in ovary and endometrium and the expression level of miRNAs based on the read counts

| Number of miRNAs | miRNA expression level | | |
| --- | --- | --- | --- |
|  | European mouflon | Finnsheep | Total |
| Ovary (373) |  |  |  |
| Sheep miRNAs (89) | 27991952 (68) | 47885007 (73) | 75876959 |
| Conserved miRNAs (174) | 1496931 (123) | 8087135 (121) | 9584066 |
| Novel miRNAs (110) | 2299 (24) | 13748 (90) | 16047 |
| Endometrium (192) |  |  |  |
| Sheep miRNAs (59) | 5049370 (47) | 4421842 (48) | 9471212 |
| Conserved miRNAs (104) | 523455 (76) | 419381 (81) | 942836 |
| Novel miRNAs (29) | 728 (13) | 666 (21) | 1394 |

The figures beyond the parentheses represent read counts, and the figures in the parentheses represent the number of miRNAs.

**Table S5. Summary information of the 122 novel miRNAs in all the 15 samples**

| No. | Mature sequence | Chr. | Strand |
| --- | --- | --- | --- |
| novel-1 | aaaaaaccgagugaacuuuuug | 12 | - |
| novel-2 | aaaaacccgaacgagcucuuugg | X | - |
| novel-3 | aaaaaccugaacgaacucuuugg | 3 | - |
| novel-4 | aaaaaguuccuuuggguuuucu | 2 | + |
| novel-5 | aaaacccgaacgagcucuuuggc | X | - |
| novel-6 | aaaaccuugaugaacuuuuuga | 4 | - |
| novel-7 | aaaacucugaaugaacuuuaug | 12 | - |
| novel-8 | aaaaguucauuuggguuguuccu-12 | 12 | - |
| novel-9 | aaaaguucauuuggguuguuccu-13 | 13 | - |
| novel-10 | aaaaguucauuuggguuguuccu-X | X | - |
| novel-11 | aaaaguuuguuuggguuuuucc | X | + |
| novel-12 | aaacccgaacgaacuuuugggc | 18 | - |
| novel-13 | aaacccgaaugaacuucuuugc | 9 | - |
| novel-14 | aaacccugaacgaaggucuugg | 12 | - |
| novel-15 | aaacucgugucugauucguua- | 4 | - |
| novel-16 | aaacucgugucugauucguua+ | 4 | + |
| **novel-17** | **aaggccgagaggacugagcccu** | **1** | **+** |
| novel-18 | aaguggagccagaaaccuugcc | 20 | - |
| **novel-19** | **aaguggagccagaaaccuugcca** | **20** | **-** |
| novel-20 | aauggugcuuuuuugugaag | X | - |
| novel-21 | aauggugcuuuuuugugaaga | X | - |
| novel-22 | aauguacuuguggaguuggaga | 7 | + |
| novel-23 | aauugucuccgucuuuucagaa | 5 | - |
| novel-24 | acaaagaggacacggcggaag | 3 | + |
| novel-25 | acaaagaggacacggcggaagu | 3 | + |
| novel-26 | acaucucagucacuuucuguu | 7 | + |
| novel-27 | acccuggugaccugugacagag | 6 | - |
| novel-28 | accggcacauuacccgucugacg | 11 | + |
| novel-29 | acgcgcugccuuugagcccccg | 3 | + |
| novel-30 | acgugagcaggauguagaagu | 2 | + |
| novel-31 | acugcccuucugccccugccag | 1 | - |
| novel-32 | acugcuuuaaaaccuuugacag | 23 | - |
| novel-33 | agcagagcgagcaggacagugc | 3 | - |
| novel-34 | aggcccggcucucaggacugc | 14 | - |
| **novel-35** | **aggcccggcucucaggacugcu** | **14** | **-** |
| novel-36 | auaaguucauucggaguugucc | 12 | - |
| novel-37 | aucugaacaaacuuuuuggaca | 21 | - |
| novel-38 | auggagauggugccuacugcaug | 4 | - |
| novel-39 | augugaaauuaccaguauuug | 7 | + |
| novel-40 | augugaaauuaccaguauuugu | 7 | + |
| **novel-41** | **auuggcacgucuuggaauga** | **X** | **-** |
| novel-42 | auuggcacgucuuggaaugaa | X | - |
| novel-43 | caaaaccugaacgaacuuuuugu | 3 | - |
| novel-44 | caaaugaacuuuuuggucaacc | 15 | + |
| novel-45 | caacacgcgucagucaggcucu | 15 | + |
| novel-46 | caagaaguucguuuggguuuu | 5 | - |
| novel-47 | caaggcaggcagaguagggcugu | 6 | - |
| novel-48 | cacagguuuugcuuccaucacug | 13 | + |
| novel-49 | cacauggaguugcuguuacaau | 12 | + |
| novel-50 | cagcaacuccugacaacuaga | 26 | - |
| novel-51 | cauccuggauccucuuaaccucu | 24 | - |
| novel-52 | cccggcccucuucucucgccagg | 12 | + |
| novel-53 | ccgcccucagugugaccgcggga | 1 | + |
| novel-54 | ccgcccucagugugaccgcgggc | 1 | - |
| **novel-55** | **ccgggccugggauccucuaugc** | **5** | **-** |
| novel-56 | ccggggaaagcaggagugagggu | 4 | - |
| novel-57 | ccucguccugcuuuccccaca | 4 | - |
| **novel-58** | **ccucucucgguuagcuccauaga** | **26** | **+** |
| novel-59 | cgagguucggcgauccgggaacuu | 3 | + |
| **novel-60** | **cgcccucugcccgugucgccagg** | **5** | **-** |
| novel-61 | cgcuucgccccugccgcccaagu | 4 | - |
| novel-62 | cuccccuucccccaccucacagu | 11 | - |
| novel-63 | cucguccugcuuuccccacagg | 4 | - |
| novel-64 | cugaaaagacucagacaauugcu | 5 | - |
| **novel-65** | **cugcugagccgucgcuuucacu** | **4** | **+** |
| novel-66 | gaaaaguucauucggguuuuu | 13 | - |
| novel-67 | gaaaaguucauucggguuuuuca | X | - |
| novel-68 | gaaagguucauuuggguuuuu | 10 | - |
| novel-69 | gaaaguguaaggcaaggucuggu | 15 | - |
| novel-70 | gaagaaucugaaugaacauuu | 1 | + |
| novel-71 | gaagguuuguuuggguuuuucu | 10 | - |
| novel-72 | gaccaaugaugaagacuagu | 18 | + |
| novel-73 | gaggguuuggguuaacaucug | 3 | - |
| novel-74 | gaugaggcucagcgagcccug | 17 | + |
| novel-75 | gcaaggugcuguacccucuuggcu | 15 | + |
| **novel-76** | **gcuccccacccccuaccuccacc** | **3** | **+** |
| novel-77 | gcugaggcugcucaggcuguga | 14 | - |
| **novel-78** | **ggaggguuuggguuaacaucug** | **3** | **-** |
| novel-79 | guggacuucccugguagcucagcu | 2 | + |
| novel-80 | uaacugaguccuuuggaaaau | X | + |
| novel-81 | uacggaggggagccagggagcacu | 3 | - |
| novel-82 | uagagaagcgcugggggaaagc | 22 | + |
| novel-83 | uauucccagcuugcauuccacu | 22 | + |
| novel-84 | ucacugggcauccucugcuuu | 18 | - |
| novel-85 | ucacugggcauccucugcuuuau | 18 | - |
| novel-86 | uccccgagucccuggcgugcacu | 1 | + |
| novel-87 | uccccgagucccuggcgugcacug | 1 | + |
| novel-88 | ucccgggcuggaggagucugca | 14 | + |
| novel-89 | ucccgggcuggaggagucugcag | 14 | + |
| **novel-90** | **ucccugucuucaauccuguagu** | **14** | **-** |
| **novel-91** | **uccuguuaaucucuccccaga** | **2** | **-** |
| novel-92 | ucggacacgccugaggggacu | 4 | - |
| **novel-93** | **ucugagacaggagggcaggugg** | **7** | **-** |
| novel-94 | ucugagacaggagggcagguggu | 7 | - |
| novel-95 | ucuggaggggcagaaggagaag | X | - |
| novel-96 | ugaaaaguucauucggguuuuu | 13 | - |
| novel-97 | ugauguuuaucuggauccucaga | 3 | - |
| novel-98 | ugcaguagcguccauccucagac | 4 | - |
| novel-99 | ugcuccccuucccccaccucacagu | 11 | - |
| **novel-100** | **uggagccggagcugguuagcgg** | **1** | **-** |
| novel-101 | uggagccggagcugguuagcggc | 1 | - |
| novel-102 | uggccacuugcucagcccgggc | 10 | + |
| novel-103 | uggcugcauggcuguuggacc | 11 | - |
| **novel-104** | **uguacucugggacucggguguca** | **1** | **+** |
| novel-105 | ugugacugcuagaaccgcuccu | 5 | + |
| novel-106 | ugugacugcuagaaccgcuccug | 5 | + |
| **novel-107** | **ugugacugcuagaaccgcuccugc** | **5** | **+** |
| novel-108 | ugugcagaagcccugagucca | 3 | - |
| novel-109 | uguggagugacugucagaugcag | 1 | + |
| novel-110 | uucacuugcucaucgugucuga | 1 | + |
| novel-111 | uucagccguguccucuuugcga | 3 | + |
| **novel-112** | **uuccggucgcugugcucucgcc** | **3** | **+** |
| novel-113 | uuccuagacccugcccugcgc | 3 | + |
| **novel-114** | **uucucagcgugucccuccuacc** | **17** | **-** |
| novel-115 | uucuucccacgcguguccgcag | 5 | + |
| novel-116 | uugaaagggaaugacagcaggg | 3 | - |
| novel-117 | uugcuauugucgucugaugcacc | 11 | + |
| novel-118 | uugcucaguugugucugacuguu | 25 | + |
| **novel-119** | **uugcucccuccacacuuccaga** | **11** | **-** |
| novel-120 | uuggacacuucaguacugcuac | 18 | - |
| novel-121 | uuguugucaggagucacugcu | 26 | - |
| novel-122 | uuugcaccucugagaguggagu | 5 | + |

The 20 miRNAs in bold type were also identified as novel miRNAs in our previous study of Hu *et al*. (2016).

**Table S6. The number of sheep and conserved miRNAs distributed on sheep chromosomes**

| Chr. | Ovary | | Endometrium | |
| --- | --- | --- | --- | --- |
|  | European mouflon | Finnsheep | European mouflon | Finnsheep |
| 1 | 15 | 15 | 12 | 11 |
| 2 | 15 | 15 | 11 | 11 |
| 3 | 19 | 15 | 7 | 9 |
| 4 | 8 | 6 | 5 | 7 |
| 5 | 9 | 9 | 9 | 9 |
| 6 | 3 | 2 | 1 | 1 |
| 7 | 6 | 5 | 5 | 5 |
| **8** | 0 | 0 | 0 | 0 |
| 9 | 4 | 7 | 5 | 5 |
| 10 | 7 | 7 | 3 | 6 |
| 11 | 16 | 9 | 7 | 7 |
| 12 | 10 | 12 | 6 | 6 |
| 13 | 5 | 3 | 2 | 3 |
| 14 | 3 | 6 | 2 | 3 |
| 15 | 5 | 6 | 5 | 4 |
| 16 | 3 | 1 | 3 | 1 |
| 17 | 0 | 1 | 0 | 0 |
| **18** | 37 | 40 | 14 | 18 |
| 19 | 6 | 4 | 4 | 3 |
| 20 | 1 | 2 | 2 | 1 |
| 21 | 5 | 5 | 2 | 2 |
| 22 | 2 | 2 | 1 | 1 |
| 23 | 2 | 1 | 1 | 1 |
| 24 | 5 | 5 | 4 | 5 |
| 25 | 0 | 2 | 0 | 0 |
| 26 | 2 | 3 | 0 | 1 |
| **X** | 28 | 32 | 17 | 23 |

**Table S7. The top expressed genes (FPKM > 3000) for European mouflon and Finnsheep mRNAs based on the sheep reference genome Oar v.4.0**

| Ovary | | Endometrium | |
| --- | --- | --- | --- |
| European mouflon | Finnsheep | European mouflon | Finnsheep |
| *RPL23A,RPLP1,RPS29,RPS24,RPS11,RPL31,RPS12,RPL35A, RPS18,RPS17,RPS27A,RPS15A,RPS27,RPL7A,RPL13A,ATP6,ATP8,MGP,OVAR-DRB3,B3GALT4* | *RPS24, RPS8,RPL23A, RPS12,RPS15A,RPLP1,RPL35A,ND3,TPT1* | *RPL23A*,*RPS8,RPLP1,RPS24,RPS12*,*RPS2,RPL31,RPS11,RPS18,RPS27,RPL32,RPL35A,RPS15A,RPL13A,RPL23,RPL39,RPS17,RPL35,RPS20,RPL26,RPS2,RPL4,ATP6*,*ATP8*,*COX1*,*COX3,B3GALT4, TPT1* | *RPS29,ATP6*,*ATP8*,*COX3*,*COX2*,*MGP*,*ND4*,*ND4L*,*CYTB*,*ND1*,*ND2,WFDC2,ND3,IL27,NUPR1,ND6,B2M,TPT1* |

**Table S8.** **Summary information of the top abundantly expressed sheep and conserved miRNAs**

| Profile | miRNA | Sequences (5'-3') | Reference species | Chr. | Strand | baseMean |
| --- | --- | --- | --- | --- | --- | --- |
| Finnsheep ovary | **oar-miR-10b** | uacccuguagaaccgaauuugu | OAR | 2 | － | 1034848 |
|  | **oar-miR-143** | ugagaugaagcacuguagcu | OAR | 5 | ＋ | 484853 |
|  | **oar-miR-26a** | uucaaguaauccaggauaggcu | OAR | 3 | ＋ | 458010 |
|  | oar-miR-181a | aacauucaacgcugucggugag | OAR | 3 | － | 247360 |
|  | oar-let-7a | ugagguaguagguuguauaguu | OAR | 15 | － | 168653 |
|  | **oar-miR-191** | caacggaaucccaaaagcagcug | OAR | 19 | ＋ | 106561 |
|  | **oar-miR-22-3p** | aagcugccaguugaagaacugu | OAR | 11 | － | 103904 |
|  | oar-let-7c | cuguacaaccuucuagcuuucc | OAR | 1 | － | 82949.5 |
|  | **oar-let-7f** | ugagguaguagauuguauaguu | OAR | 2 | － | 73651.6 |
|  | **miR-27b** | uucacaguggcuaaguucug | BTA | 2 | － | 61265.2 |
| European mouflon ovary | **oar-miR-10b** | uacccuguagaaccgaauuugu | OAR | 2 | － | 3629611 |
|  | **oar-miR-143** | ugagaugaagcacuguagcu | OAR | 5 | ＋ | 1930668 |
|  | **oar-miR-26a** | uucaaguaauccaggauaggcu | OAR | 3 | ＋ | 689868 |
|  | oar-miR-148a | ucagugcacuacagaacuuugu | OAR | 4 | ＋ | 280748 |
|  | **miR-27b** | uucacaguggcuaaguucug | BTA | 2 | － | 140810 |
|  | **oar-let-7f** | ugagguaguagauuguauaguu | OAR | 2 | － | 135242 |
|  | **oar-miR-191** | caacggaaucccaaaagcagcug | OAR | 19 | ＋ | 67241.7 |
|  | oar-miR-199a-3p | acaguagucugcacauugguu | OAR | 12 | － | 65326.1 |
|  | oar-miR-99a | aacccguagauccgaucuugu | OAR | 1 | － | 58744.3 |
|  | **oar-miR-22-3p** | aagcugccaguugaagaacugu | OAR | 11 | － | 46073.6 |
| Finnsheep endometrium | *oar-miR-10b* | uacccuguagaaccgaauuugu | OAR | 2 | － | 50560.4 |
|  | miR-378 | acuggacuuggagucagaaggc | BTA | 5 | ＋ | 16545.7 |
|  | oar-miR-19b | ugugcaaauccaugcaaaacug | OAR | 10 | + | 3384.19 |
|  | *miR-100* | aacccguagauccgaacuugu | BTA | 15 | － | 681.543 |
|  | *miR-34c-5p* | aggcaguguaguuagcugauugc | HAS | 15 | ＋ | 168.738 |
| European mouflon endometrium | *oar-miR-10b* | uacccuguagaaccgaauuugu | OAR | 2 | － | 713956 |
|  | oar-let-7a | ugagguaguagguuguauaguu | OAR | 15 | - | 49988.2 |
|  | miR-186 | caaagaauucuccuuuugggcu | BTA | 1 | - | 44282.8 |
|  | *miR-100* | aacccguagauccgaacuugu | BTA | 15 | － | 6649 |
|  | miR-204 | uucccuuugucauccuaugccu | BTA | 2 | + | 6014.44 |
|  | oar-miR-23b | aucacauugccagggauuaccacg | OAR | 2 | - | 4929.22 |
|  | *miR-34c-5p* | aggcaguguaguuagcugauugc | HAS | 15 | ＋ | 4484.18 |
|  | miR-449a | uggcaguguauuguuagcuggu | BTA | 16 | + | 4133.34 |
|  | oar-miR-103 | agcagcauuguacagggcuauga | OAR | 16 | - | 3586.78 |
|  | miR-101-3p | guacaguacugugauaacu | GGA | 1 | - | 3246.66 |

OAR = *Ovis aries*; BTA = *Bos Taurus*.

The common ovarian miRNAs between Finnsheep and European mouflon are shown in bold.

The common endometrial miRNAs between Finnsheep and European mouflon are indicated by italics.

**Table S9.** Summary information of the specifically expressed sheep and conserved miRNAs

| Profile | miRNA | Sequences (5'-3') | Reference species | Chr. | Strand | baseMean |
| --- | --- | --- | --- | --- | --- | --- |
| Finnsheep ovary | miR-181a | aacauucaacgcugucggugag | OAR | 3 | － | 247359.982 |
|  | let-7a | ugagguaguagguuguauaguu | OAR | 15 | － | 168653.417 |
|  | miR-30a-5p | uguaaacauccucgacugga | OAR | 9 | ＋ | 38751.9493 |
|  | miR-181b | aacauucauugcugucggugggu | BTA | 3 | － | 3521.83991 |
|  | miR-424-3p | caaaacgugaggcgcugcuau | BTA | X | ＋ | 1810.62883 |
|  | miR-24-3p | uggcucaguucagcaggaac | CGR | 5 | ＋ | 639.634964 |
|  | miR-342 | ucucacacagaaaucgcacccauc | BTA | 18 | ＋ | 608.322871 |
|  | miR-769 | ugagaccuccggguucugagcu | BTA | 14 | ＋ | 401.483947 |
|  | miR-1388-3p | aucucagguucgucagcccgca | CHI | 13 | ＋ | 280.724632 |
|  | miR-3601 | ucggggaucaucaugucacgaga | BTA | X | ＋ | 114.285234 |
|  | miR-135a-3p | uguagggauggaagccaugaaa | RNO | 3 | ＋ | 64.585094 |
|  | miR-335-3p | uuuuucauuauugcuccugacc | HSA | 4 | ＋ | 60.2421038 |
| European mouflon ovary | miR-145a-3p | auuccuggaaauacuguucuu | MMU | 5 | ＋ | 6159.19212 |
|  | miR-424-5p | cagcagcaauucauguuuuga | BTA | X | ＋ | 1959.80579 |
|  | miR-148b | ucagugcaucacagaacuuugu | BTA | 3 | － | 1885.9224 |
|  | miR-142-5p | cauaaaguagaaagcacuacu | HSA | 11 | － | 1577.88613 |
|  | miR-542-3p | ugugacagauugauaacugaaa | MMU | X | ＋ | 1183.0864 |
|  | miR-450b | uuuugcaauauguuccugaau | BTA | X | ＋ | 1175.74149 |
|  | miR-155 | uuaaugcuaaucgugauaggggu | BTA | 1 | － | 769.322426 |
|  | miR-339a | ucccuguccuccaggagcuc | BTA | 24 | ＋ | 631.739746 |
|  | miR-152 | ucagugcaugacagaacuuggg | OAR | 11 | ＋ | 317.21565 |
|  | miR-1388-5p | aggacuguccaaccugagaau | BTA | 13 | ＋ | 296.981275 |
|  | miR-19a | ugugcaaaucuaugcaaaacug | BTA | 10 | ＋ | 200.491422 |
| Finnsheep endometrium | miR-9-5p | ucuuugguuaucuagcuguauga | HSA | 5 | - | 4512.47928 |
|  | miR-19b | ugugcaaauccaugcaaaacug | OAR | 10 | + | 3384.18527 |
|  | miR-128 | ucacagugaaccggucucuuu | BTA | 2 | - | 1885.02143 |
| European mouflon endometrium | let-7a | ugagguaguagguuguauaguu | OAR | 15 | - | 49988.1953 |
|  | miR-186 | caaagaauucuccuuuugggcu | BTA | 1 | - | 44282.8491 |
|  | miR-204 | uucccuuugucauccuaugccu | BTA | 2 | + | 6014.44446 |
|  | miR-23b | aucacauugccagggauuaccacg | OAR | 2 | - | 4929.216 |
|  | miR-449a | uggcaguguauuguuagcuggu | BTA | 16 | + | 4133.34418 |
|  | miR-103 | agcagcauuguacagggcuauga | OAR | 16 | - | 3586.78161 |
|  | miR-101-3p | guacaguacugugauaacu | GGA | 1 | - | 3246.66063 |
|  | miR-34b-3p | aaucacuaguuccacugccauc | CHI | 15 | + | 931.243347 |
|  | miR-107 | agcagcauuguacagggcuau | OAR | 22 | - | 885.555454 |

OAR = *Ovis aries*; BTA = *Bos Taurus*.

**Table S12.** Eight overlapping genes between 192 ovarian DEGs and reported reproductive QTLs

| Gene ID | log2(fold_change) | q_value | Chromosome | Gene start site | Gene end site | QTL start site | QTL end site | Traits |
| --- | --- | --- | --- | --- | --- | --- | --- | --- |
| NME9 | -4.90398 | 0.00536836 | 1 | 248697522 | 248723374 | 197308986 | 271106485 | Reproductive seasonality |
| LRRIQ4 | -3.83833 | 0.00750672 | 1 | 215412673 | 215434492 | 197308986 | 271106485 | Reproductive seasonality |
| SPATC1L | -3.46112 | 0.00439394 | 1 | 263812791 | 263830695 | 197308986 | 271106485 | Reproductive seasonality |
| PRDM15 | -2.67899 | 0.00160786 | 1 | 259967416 | 260029716 | 197308986 | 271106485 | Reproductive seasonality |
| PEX5L | -2.50138 | 0.0048628 | 1 | 205151746 | 205364186 | 197308986 | 271106485 | Reproductive seasonality |
| COL6A6 | -2.15767 | 0.0048628 | 1 | 269444011 | 269614155 | 197308986 | 271106485 | Reproductive seasonality |
| RARRES1 | -2.0974 | 0.00374218 | 1 | 226751278 | 226791676 | 197308986 | 271106485 | Reproductive seasonality |
| SPOCK1 | -2.79607 | 0.00536836 | 5 | 45664388 | 46009097 | 45779628 | 45779668 | Total lambs born |

**Table S13.** Seventy-four overlapping genes between 1065 endometrial DEGs and reported reproductive QTLs

| Gene ID | log2(fold_change) | q_value | Chromosome | Gene start site | Gene end site | QTL start site | QTL end site | Traits |
| --- | --- | --- | --- | --- | --- | --- | --- | --- |
| SUCNR1 | -6.26583 | 0.00357011 | 1 | 233966987 | 233977682 | 197308986 | 271106485 | Reproductive seasonality |
| UPK1B | -6.22704 | 0.000779626 | 1 | 182123725 | 182155017 | 168602638 | 197308986 | Reproductive seasonality |
| MUC4 | -5.72778 | 0.00267452 | 1 | 188582359 | 188630422 | 168602638 | 197308986 | Reproductive seasonality |
| IGSF10 | -5.10457 | 0.000779626 | 1 | 234544940 | 234571872 | 197308986 | 271106485 | Reproductive seasonality |
| RASL11B | -4.84315 | 0.0020136 | 6 | 68412630 | 68417112 | 68295117 | 68445447 | Total lambs born |
| DGKG | -4.83005 | 0.000779626 | 1 | 198927557 | 199155167 | 197308986 | 271106485 | Reproductive seasonality |
| COL18A1 | -4.77051 | 0.000779626 | 1 | 263182392 | 263236931 | 197308986 | 271106485 | Reproductive seasonality |
| GMNC | -4.71946 | 0.00222425 | 1 | 194501709 | 194512472 | 168602638 | 197308986 | Reproductive seasonality |
| RBP1 | -4.55763 | 0.00222425 | 1 | 247420622 | 247448039 | 197308986 | 271106485 | Reproductive seasonality |
| ROPN1 | -4.33428 | 0.00140663 | 1 | 186463643 | 186493960 | 168602638 | 197308986 | Reproductive seasonality |
| CLDN11 | -4.03638 | 0.00140663 | 1 | 214888272 | 214903261 | 197308986 | 271106485 | Reproductive seasonality |
| CLSTN3 | -3.97539 | 0.00311413 | 3 | 206993068 | 207024989 | 190634661 | 208095372 | Reproductive seasonality |
| CCDC39 | -3.6758 | 0.00267452 | 1 | 204404074 | 204460692 | 197308986 | 271106485 | Reproductive seasonality |
| IL20RB | -3.66232 | 0.000779626 | 1 | 250083067 | 250120265 | 197308986 | 271106485 | Reproductive seasonality |
| COL6A1 | -3.34305 | 0.000779626 | 1 | 263618448 | 263638673 | 197308986 | 271106485 | Reproductive seasonality |
| FOXL2 | -3.16707 | 0.0080546 | 1 | 248088730 | 248095868 | 197308986 | 271106485 | Reproductive seasonality |
| ITGB5 | -2.98939 | 0.000779626 | 1 | 187307053 | 187425351 | 168602638 | 197308986 | Reproductive seasonality |
| TMPRSS2 | -2.90767 | 0.000779626 | 1 | 259602440 | 259651515 | 197308986 | 271106485 | Reproductive seasonality |
| COL6A2 | -2.87204 | 0.000779626 | 1 | 263766217 | 263783506 | 197308986 | 271106485 | Reproductive seasonality |
| C1S | -2.86039 | 0.000779626 | 3 | 207131511 | 207142927 | 190634661 | 208095372 | Reproductive seasonality |
| P3H3 | -2.84853 | 0.00267452 | 3 | 207337861 | 207351697 | 190634661 | 208095372 | Reproductive seasonality |
| LRRC3 | -2.82443 | 0.0200887 | 1 | 262365694 | 262368775 | 197308986 | 271106485 | Reproductive seasonality |
| TNFSF10 | -2.61953 | 0.00939318 | 1 | 212908419 | 212927729 | 197308986 | 271106485 | Reproductive seasonality |
| SLC19A1 | -2.49729 | 0.00629611 | 1 | 263245238 | 263269967 | 197308986 | 271106485 | Reproductive seasonality |
| CORO1A | -2.45375 | 0.00222425 | 24 | 26140723 | 26146232 | 20640520 | 34053668 | Reproductive seasonality |
| CCDC80 | -2.43316 | 0.00443959 | 1 | 175553988 | 175589570 | 168602638 | 197308986 | Reproductive seasonality |
| TNIK | -2.40426 | 0.000779626 | 1 | 213937866 | 214341267 | 197308986 | 271106485 | Reproductive seasonality |
| SCNN1B | -2.33943 | 0.00357011 | 24 | 21058908 | 21085025 | 20640520 | 34053668 | Reproductive seasonality |
| FSTL1 | -2.24159 | 0.000779626 | 1 | 183269841 | 183321812 | 168602638 | 197308986 | Reproductive seasonality |
| EPHB3 | -2.08362 | 0.00666014 | 1 | 200666918 | 200686431 | 197308986 | 271106485 | Reproductive seasonality |
| HSPA4L | 2.04091 | 0.00999536 | 17 | 29545468 | 29602538 | 14434392 | 33120992 | Reproductive seasonality |
| MANSC1 | 2.047 | 0.00267452 | 3 | 202095737 | 202119637 | 190634661 | 208095372 | Reproductive seasonality |
| MX1 | 2.15422 | 0.00443959 | 1 | 259561625 | 259596486 | 197308986 | 271106485 | Reproductive seasonality |
| MECOM | 2.20037 | 0.00357011 | 1 | 215572144 | 216202969 | 197308986 | 271106485 | Reproductive seasonality |
| GRAMD1C | 2.20547 | 0.000779626 | 1 | 176767015 | 176813159 | 168602638 | 197308986 | Reproductive seasonality |
| PPP1R2 | 2.22891 | 0.00311413 | 1 | 190190426 | 190211297 | 168602638 | 197308986 | Reproductive seasonality |
| ATP1B3 | 2.2489 | 0.000779626 | 1 | 244902124 | 244937358 | 197308986 | 271106485 | Reproductive seasonality |
| CPEB4 | 2.26362 | 0.000779626 | 16 | 5641121 | 5714264 | 66143 | 6074273 | Reproductive seasonality |
| WBSCR27 | 2.27073 | 0.00140663 | 24 | 33375333 | 33379654 | 20640520 | 34053668 | Reproductive seasonality |
| CLEC2D | 2.33335 | 0.00872515 | 3 | 204967197 | 204982375 | 190634661 | 208095372 | Reproductive seasonality |
| GK5 | 2.36809 | 0.00443959 | 1 | 244589081 | 244666617 | 197308986 | 271106485 | Reproductive seasonality |
| ZDHHC23 | 2.40161 | 0.000779626 | 1 | 176813812 | 176829801 | 168602638 | 197308986 | Reproductive seasonality |
| PPM1L | 2.40698 | 0.000779626 | 1 | 224306283 | 224636269 | 197308986 | 271106485 | Reproductive seasonality |
| EPS8 | 2.44463 | 0.000779626 | 3 | 198797630 | 198924833 | 190634661 | 208095372 | Reproductive seasonality |
| PANK3 | 2.60751 | 0.000779626 | 16 | 327034 | 350323 | 66143 | 6074273 | Reproductive seasonality |
| SIK1 | 2.79765 | 0.000779626 | 1 | 261425727 | 261434810 | 197308986 | 271106485 | Reproductive seasonality |
| ILDR1 | 3.03181 | 0.000779626 | 1 | 184577171 | 184613755 | 168602638 | 197308986 | Reproductive seasonality |
| KNG1 | 3.09343 | 0.000779626 | 1 | 198549937 | 198575210 | 197308986 | 271106485 | Reproductive seasonality |
| LRRC34 | 3.17853 | 0.000779626 | 1 | 215434671 | 215454124 | 197308986 | 271106485 | Reproductive seasonality |
| LSS | 3.20137 | 0.000779626 | 1 | 263836789 | 263868476 | 197308986 | 271106485 | Reproductive seasonality |
| SCNN1A | 3.26252 | 0.00267452 | 3 | 207717531 | 207743132 | 190634661 | 208095372 | Reproductive seasonality |
| NUDT16 | 3.34728 | 0.000779626 | 1 | 256494474 | 256496103 | 197308986 | 271106485 | Reproductive seasonality |
| DBR1 | 3.38571 | 0.000779626 | 1 | 248846086 | 248858539 | 197308986 | 271106485 | Reproductive seasonality |
| SLCO1B3 | 3.3881 | 0.000779626 | 3 | 193447368 | 193531240 | 190634661 | 208095372 | Reproductive seasonality |
| QPRT | 3.46649 | 0.000779626 | 24 | 26499856 | 26515340 | 20640520 | 34053668 | Reproductive seasonality |
| USP31 | 3.85319 | 0.000779626 | 24 | 20783943 | 20867952 | 20640520 | 34053668 | Reproductive seasonality |
| ARHGDIB | 3.86317 | 0.000779626 | 3 | 199552992 | 199572544 | 190634661 | 208095372 | Reproductive seasonality |
| ATP13A4 | 3.87204 | 0.000779626 | 1 | 191980292 | 192109018 | 168602638 | 197308986 | Reproductive seasonality |
| SLCO1A2 | 3.90409 | 0.000779626 | 3 | 193320587 | 193433818 | 190634661 | 208095372 | Reproductive seasonality |
| C1H3orf70 | 3.97811 | 0.000779626 | 1 | 200047781 | 200165750 | 197308986 | 271106485 | Reproductive seasonality |
| LRRC31 | 4.18615 | 0.000779626 | 1 | 215383762 | 215412519 | 197308986 | 271106485 | Reproductive seasonality |
| GMPR | 4.23247 | 0.000779626 | 20 | 39675025 | 39736401 | 38448827 | 40113055 | Reproductive seasonality |
| PIK3C2G | 4.30447 | 0.00400279 | 3 | 195922763 | 196393238 | 190634661 | 208095372 | Reproductive seasonality |
| KCNJ8 | 4.32015 | 0.000779626 | 3 | 192966780 | 192973521 | 190634661 | 208095372 | Reproductive seasonality |
| NOCT | 4.44617 | 0.000779626 | 17 | 18233600 | 18255999 | 14434392 | 33120992 | Reproductive seasonality |
| ATP13A5 | 4.57831 | 0.000779626 | 1 | 192134767 | 192252365 | 168602638 | 197308986 | Reproductive seasonality |
| PSPH | 4.80876 | 0.000779626 | 24 | 27667950 | 27686029 | 20640520 | 34053668 | Reproductive seasonality |
| RERG | 4.84018 | 0.000779626 | 3 | 199279379 | 199420206 | 190634661 | 208095372 | Reproductive seasonality |
| BCL2L14 | 4.86742 | 0.000779626 | 3 | 202364759 | 202407034 | 190634661 | 208095372 | Reproductive seasonality |
| C1H3orf80 | 4.89628 | 0.00443959 | 1 | 225128790 | 225129919 | 197308986 | 271106485 | Reproductive seasonality |
| STYK1 | 5.05218 | 0.000779626 | 3 | 203605210 | 203629368 | 190634661 | 208095372 | Reproductive seasonality |
| BDH1 | 5.20152 | 0.000779626 | 1 | 190048196 | 190086503 | 168602638 | 197308986 | Reproductive seasonality |
| WBSCR17 | 5.30314 | 0.00267452 | 24 | 29052183 | 29480276 | 20640520 | 34053668 | Reproductive seasonality |
| GRM7 | 7.922 | 0.000779626 | 19 | 18570527 | 19471905 | 19011956 | 19012056 | Reproductive seasonality |

**Table S14.** Significantly enriched GO terms and KEGG pathways for the differential expressed genes (DEGs) in the comparison of ovarian mRNAs between European mouflon and Finnsheep

| **GO terms** | **Number** | **Cluster**  **of**  **genes** | **Total genes** | **Percentage**  **(%)** | | | ***P*-value** | **Genes** |
| --- | --- | --- | --- | --- | --- | --- | --- | --- |
| **Biological process** |  | | | | | | | |
| negative regulation of fertilization | GO:0060467 | 3 | 194 | 1.55 | | | 3.03E-04 | *ASTL, ZP2, ZP4* |
| binding of sperm to zona pellucida | GO:0007339 | 4 | 194 | 2.06 | | | 0.001546 | *ASTL, ZP2, ZP3, ZP4* |
| sperm-egg recognition | GO:0035036 | 4 | 194 | 2.06 | | | 0.00222 | *ASTL, ZP2, ZP3, ZP4* |
| ovarian follicle development | GO:0001541 | 4 | 194 | 2.06 | | | 0.003366 | *INHBA, ZP3, NOBOX, SOHLH1* |
| humoral immune response | GO:0006959 | 5 | 194 | 2.58 | | | 0.003753 | *C1QA, ZP3, ZP4, C4BPA, SPON2* |
| cell-cell recognition | GO:0009988 | 4 | 194 | 2.06 | | | 0.004052 | *ASTL, ZP2, ZP3, ZP4* |
| humoral immune response mediated by circulating immunoglobulin | GO:0002455 | 3 | 194 | 1.55 | | | 0.004341 | *C1QA, ZP3, C4BPA* |
| female gamete generation | GO:0007292 | 5 | 194 | 2.58 | | | 0.0052 | *INHBA, TDRD5, ZP3, PABPC1L, SOHLH1* |
| sex differentiation | GO:0007548 | 7 | 194 | 3.61 | | | 0.006011 | *ATRX, INHBA, AMH, CYP17A1, ZP3, NOBOX, SOHLH1* |
| gonad development | GO:0008406 | 6 | 194 | 3.09 | | | 0.009021 | *ATRX, INHBA, AMH, ZP3, NOBOX, SOHLH1* |
| development of primary sexual characteristics | GO:0045137 | 6 | 194 | 3.09 | | | 0.009317 | *ATRX, INHBA, AMH, ZP3, NOBOX, SOHLH1* |
| ovulation cycle process | GO:0022602 | 4 | 194 | 2.06 | | | 0.009912 | *INHBA, ZP3, NOBOX, SOHLH1* |
| regulation of reproductive process | GO:2000241 | 5 | 194 | 2.58 | | | 0.010771 | *INHBA, ASTL, ZP2, ZP3, ZP4* |
| ovulation cycle | GO:0042698 | 4 | 194 | 2.06 | | | 0.011197 | *INHBA, ZP3, NOBOX, SOHLH1* |
| oogenesis | GO:0048477 | 4 | 194 | 2.06 | | | 0.016423 | *TDRD5, ZP3, PABPC1L, SOHLH1* |
| sexual reproduction | GO:0019953 | 10 | 194 | 5.15 | | | 0.020077 | *ATRX, INHBA, TDRD5, ASTL, ZP2, ZP3, SERPINA5, ZP4, PABPC1L, SOHLH1* |
| complement activation, classical pathway | GO:0006958 | 2 | 194 | 1.03 | | | 0.020163 | *C1QA, C4BPA* |
| prevention of polyspermy | GO:0060468 | 2 | 194 | 1.03 | | | 0.020163 | *ASTL, ZP2* |
| negative regulation of binding of sperm to zona pellucida | GO:2000360 | 2 | 194 | 1.03 | | | 0.020163 | *ASTL, ZP4* |
| regulation of binding of sperm to zona pellucida | GO:2000359 | 2 | 194 | 1.03 | | | 0.020163 | *ASTL, ZP4* |
| female gonad development | GO:0008585 | 4 | 194 | 2.06 | | | 0.020862 | *INHBA, ZP3, NOBOX, SOHLH1* |
| development of primary female sexual characteristics | GO:0046545 | 4 | 194 | 2.06 | | | 0.020862 | *INHBA, ZP3, NOBOX, SOHLH1* |
| cell recognition | GO:0008037 | 4 | 194 | 2.06 | | | 0.024842 | *ASTL, ZP2, ZP3, ZP4* |
| single fertilization | GO:0007338 | 4 | 194 | 2.06 | | | 0.025896 | *ASTL, ZP2, ZP3, ZP4* |
| female sex differentiation | GO:0046660 | 4 | 194 | 2.06 | | | 0.031524 | *INHBA, ZP3, NOBOX, SOHLH1* |
| purine ribonucleoside triphosphate biosynthetic process | GO:0009206 | 3 | 194 | 1.55 | | | 0.034571 | *ATP5G1, ATP5I, NME9* |
| purine nucleoside triphosphate biosynthetic process | GO:0009145 | 3 | 194 | 1.55 | | | 0.0368 | *ATP5G1, ATP5I, NME9* |
| purine ribonucleoside triphosphate metabolic process | GO:0009205 | 5 | 194 | 2.58 | | | 0.03897 | *TAZ, ALDOB, ATP5G1, ATP5I, NME9* |
| ribonucleoside triphosphate biosynthetic process | GO:0009201 | 3 | 194 | 1.55 | | | 0.039083 | *ATP5G1, ATP5I, NME9* |
| positive regulation of reproductive process | GO:2000243 | 3 | 194 | 1.55 | | | 0.039083 | *INHBA, ZP3, ZP4* |
| ribonucleoside triphosphate metabolic process | GO:0009199 | 5 | 194 | 2.58 | | | 0.040913 | *TAZ, ALDOB, ATP5G1, ATP5I, NME9* |
| oocyte development | GO:0048599 | 3 | 194 | 1.55 | | | 0.043805 | *TDRD5, ZP3, PABPC1L* |
| multi-organism reproductive process | GO:0044703 | 10 | 194 | 5.15 | | | 0.044018 | *ATRX, INHBA, TDRD5, ASTL, ZP2, ZP3, SERPINA5, ZP4, PABPC1L, SOHLH1* |
| purine nucleoside triphosphate metabolic process | GO:0009144 | 5 | 194 | 2.58 | | | 0.046006 | *TAZ, ALDOB, ATP5G1, ATP5I, NME9* |
| adaptive immune response based on somatic recombination of immune receptors built from immunoglobulin superfamily domains | GO:0002460 | 5 | 194 | 2.58 | | | 0.049225 | *C1QA, PRKCQ, IL1RL1, ZP3, C4BPA* |
| egg activation | GO:0007343 | 2 | 194 | 1.03 | | | 0.049654 | *ASTL, ZP2* |
| **Cellular component** |  | | | | | | | |
| extracellular space | GO:0005615 | | 19 | | 194 | 9.79 | 4.99E-04 | *PRKAG3, GLDN, SPOCK1, SELENBP1, DLK1, C4BPA, C1QC, MIF, RAB11FIP4, INHBA, ACSM1, F5, EREG, SERPINA5, KCP, KLK11, SERPINA1, SPON2, SPP1* |
| extracellular matrix | GO:0031012 | | 10 | | 194 | 5.15 | 0.002137453 | *EGFL7, COL6A6, ZP2, ADAMTSL2, IL1RL1, ZP3, SPOCK1, SPON2, VIT, COL8A2* |
| secretory vesicle | GO:0099503 | | 7 | | 194 | 3.61 | 0.012231919 | *GABRA2, STK31, ASTL, F5, ZP2, ZP3, SERPINA5* |
| collagen trimer | GO:0005581 | | 4 | | 194 | 2.06 | 0.012276379 | *C1QA, COL9A2, C1QC, COL8A2* |
| multivesicular body | GO:0005771 | | 3 | | 194 | 1.55 | 0.012301698 | *ZP2, ZP3, ATP13A2* |
| secretory granule | GO:0030141 | | 6 | | 194 | 3.09 | 0.012986249 | *STK31, ASTL, F5, ZP2, ZP3, SERPINA5* |
| sperm part | GO:0097223 | | 5 | | 194 | 2.58 | 0.014204439 | *STK31, ZP3, SERPINA5, SAXO1, DNAI2* |
| extracellular region | GO:0005576 | | 39 | | 194 | 20.10 | 0.03523348 | *PRKAG3, RARRES1, GLDN, ADAMTSL2, ALDOB, KIAA1324, ACP5, SELENBP1, SPOCK1, DLK1, VIT, ACAT1, C1QC, MIF, COL6A6, KRT7, SERPINA5, KLK12, KLK11, SERPINA1, SPON2, SPP1, IL1RL1, ZP4, C4BPA, DECR1, PTGFR, LCN2, RAB11FIP4, C1QA, AMH, INHBA, PDDC1, ACSM1, F5, EREG, KCP, PABPC1L, ASIP* |
| **KEGG pathway** |  | | | | | | | |
| Complement and coagulation cascades | oas04610 | | 6 | | 194 | 3.09 | 2.89E-04 | *C1QA, F5, SERPINA5, SERPINA1, C4BPA, C1QC* |
| Ovarian steroidogenesis | oas04913 | | 4 | | 194 | 2.06 | 0.007889449 | *CYP17A1, HSD3B1, CYP19, PLA2G4B* |
| MAPK signaling pathway | oas04010 | | 7 | | 194 | 3.61 | 0.011283562 | *MAP3K6, GADD45G, CACNG6, NR4A1, CACNA1E, PLA2G4B, NGF* |

**Table S15. The top 10 enriched GO terms and KEGG pathways for the differential expressed genes (DEGs) in the comparison of endometrial mRNAs between European mouflon and Finnsheep**

| **GO Term** | **GO number** | **Cluster genes** | **Total genes** | **Percentage**  **(%)** | ***P* value** |
| --- | --- | --- | --- | --- | --- |
| **Biological process** | | | | | |
| single-organism process | GO:0044699 | 623 | 1075 | 57.95 | 2.30E-12 |
| single-multicellular organism process | GO:0044707 | 333 | 1075 | 30.98 | 1.36E-11 |
| multicellular organismal process | GO:0032501 | 356 | 1075 | 33.12 | 9.99E-11 |
| system development | GO:0048731 | 259 | 1075 | 24.09 | 2.48E-10 |
| blood vessel development | GO:0001568 | 63 | 1075 | 5.86 | 4.23E-10 |
| single-organism cellular process | GO:0044763 | 559 | 1075 | 52.00 | 4.76E-10 |
| vasculature development | GO:0001944 | 65 | 1075 | 6.05 | 1.15E-09 |
| circulatory system development | GO:0072359 | 86 | 1075 | 8.00 | 1.50E-09 |
| cardiovascular system development | GO:0072358 | 86 | 1075 | 8.00 | 1.50E-09 |
| multicellular organism development | GO:0007275 | 271 | 1075 | 25.21 | 4.07E-09 |
| **Cellular process** | | | | | |
| extracellular region part | GO:0044421 | 261 | 1075 | 24.28 | 4.05E-16 |
| extracellular vesicle | GO:1903561 | 212 | 1075 | 19.72 | 4.39E-15 |
| extracellular organelle | GO:0043230 | 212 | 1075 | 19.72 | 4.62E-15 |
| extracellular region | GO:0005576 | 276 | 1075 | 25.67 | 1.16E-14 |
| extracellular exosome | GO:0070062 | 209 | 1075 | 19.44 | 1.78E-14 |
| extracellular matrix | GO:0031012 | 56 | 1075 | 5.21 | 4.45E-14 |
| vesicle | GO:0031982 | 238 | 1075 | 22.14 | 1.15E-13 |
| membrane-bounded vesicle | GO:0031988 | 229 | 1075 | 21.30 | 4.33E-13 |
| proteinaceous extracellular matrix | GO:0005578 | 40 | 1075 | 3.72 | 5.89E-10 |
| extracellular matrix component | GO:0044420 | 23 | 1075 | 2.14 | 7.59E-09 |
| **Molecular function** | | | | | |
| calcium ion binding | GO:0005509 | 56 | 1075 | 5.21 | 8.06E-06 |
| molecular function regulator | GO:0098772 | 67 | 1075 | 6.23 | 4.65E-04 |
| transmembrane receptor protein tyrosine kinase activity | GO:0004714 | 10 | 1075 | 0.93 | 0.001156 |
| anion transmembrane transporter activity | GO:0008509 | 17 | 1075 | 1.58 | 0.001487 |
| oxidoreductase activity | GO:0016491 | 54 | 1075 | 5.02 | 0.001514 |
| extracellular matrix structural constituent | GO:0005201 | 8 | 1075 | 0.74 | 0.001822 |
| transmembrane receptor protein kinase activity | GO:0019199 | 11 | 1075 | 1.02 | 0.002454 |
| enzyme regulator activity | GO:0030234 | 48 | 1075 | 4.47 | 0.002495 |
| catalytic activity | GO:0003824 | 303 | 1075 | 28.19 | 0.003895 |
| nucleotide diphosphatase activity | GO:0004551 | 4 | 1075 | 0.37 | 0.003953 |
| **KEGG pathway** | | | | | |
| Steroid biosynthesis | oas00100 | 9 | 1075 | 0.84 | 3.33E-05 |
| Biosynthesis of antibiotics | oas01130 | 29 | 1075 | 2.70 | 1.07E-04 |
| Metabolic pathways | oas01100 | 107 | 1075 | 9.95 | 2.07E-04 |
| Protein digestion and absorption | oas04974 | 19 | 1075 | 1.77 | 5.50E-04 |
| Staphylococcus aureus infection | oas05150 | 13 | 1075 | 1.21 | 8.97E-04 |
| ECM-receptor interaction | oas04512 | 14 | 1075 | 1.30 | 0.002469 |
| Arrhythmogenic right ventricular cardiomyopathy (ARVC) | oas05412 | 12 | 1075 | 1.12 | 0.002659 |
| Proteoglycans in cancer | oas05205 | 24 | 1075 | 2.23 | 0.002788 |
| MicroRNAs in cancer | oas05206 | 22 | 1075 | 2.05 | 0.00369 |
| Complement and coagulation cascades | oas04610 | 12 | 1075 | 1.12 | 0.00686 |

**Table S16. The top 10 significantly enriched GO terms for the differential expressed genes (DEGs) in the comparison of ovarian and endometrial mRNAs in European mouflon based on the sheep reference genome Oar v.4.0**

| GO Term | GO number | Cluster genes | Total genes | Percentage  (%) | *P*-value |
| --- | --- | --- | --- | --- | --- |
| **Biological process** | | | | | |
| single-multicellular organism process | GO:0044707 | 626 | 1949 | 32.12 | 2.00E-32 |
| multicellular organismal process | GO:0032501 | 672 | 1949 | 34.48 | 5.89E-32 |
| single-organism process | GO:0044699 | 1110 | 1949 | 56.95 | 2.70E-28 |
| system development | GO:0048731 | 489 | 1949 | 25.09 | 6.46E-28 |
| multicellular organism development | GO:0007275 | 514 | 1949 | 26.37 | 4.88E-26 |
| single-organism developmental process | GO:0044767 | 566 | 1949 | 29.04 | 2.39E-25 |
| anatomical structure development | GO:0048856 | 564 | 1949 | 28.94 | 5.57E-25 |
| developmental process | GO:0032502 | 574 | 1949 | 29.45 | 7.45E-25 |
| single-organism cellular process | GO:0044763 | 988 | 1949 | 50.69 | 6.12E-20 |
| vasculature development | GO:0001944 | 119 | 1949 | 6.11 | 8.65E-20 |
| **Cellular process** | | | | | |
| extracellular matrix | GO:0031012 | 104 | 1949 | 5.34 | 1.12E-29 |
| plasma membrane part | GO:0044459 | 233 | 1949 | 11.95 | 2.11E-23 |
| cell periphery | GO:0071944 | 437 | 1949 | 22.42 | 5.69E-21 |
| plasma membrane | GO:0005886 | 425 | 1949 | 21.81 | 2.37E-20 |
| proteinaceous extracellular matrix | GO:0005578 | 72 | 1949 | 3.69 | 3.43E-19 |
| intrinsic component of plasma membrane | GO:0031226 | 119 | 1949 | 6.11 | 4.02E-17 |
| integral component of plasma membrane | GO:0005887 | 112 | 1949 | 5.75 | 2.84E-16 |
| extracellular matrix component | GO:0044420 | 36 | 1949 | 1.85 | 7.76E-13 |
| extracellular region part | GO:0044421 | 396 | 1949 | 20.32 | 1.48E-12 |
| extracellular region | GO:0005576 | 429 | 1949 | 22.01 | 2.50E-12 |
| **Molecular function** | | | | | |
| calcium ion binding | GO:0005509 | 104 | 1949 | 5.34 | 1.46E-12 |
| transmembrane receptor protein tyrosine kinase activity | GO:0004714 | 20 | 1949 | 1.02 | 2.17E-08 |
| transmembrane receptor protein kinase activity | GO:0019199 | 23 | 1949 | 1.18 | 3.00E-08 |
| carbohydrate derivative binding | GO:0097367 | 235 | 1949 | 12.06 | 1.68E-07 |
| motor activity | GO:0003774 | 29 | 1949 | 1.49 | 3.65E-07 |
| microtubule motor activity | GO:0003777 | 22 | 1949 | 1.13 | 7.80E-07 |
| glycosaminoglycan binding | GO:0005539 | 30 | 1949 | 1.54 | 1.81E-06 |
| RNA polymerase II transcription factor activity, sequence-specific DNA binding | GO:0000981 | 71 | 1949 | 3.64 | 2.42E-06 |
| protein tyrosine kinase activity | GO:0004713 | 27 | 1949 | 1.39 | 5.39E-06 |
| metalloendopeptidase activity | GO:0004222 | 27 | 1949 | 1.39 | 1.18E-05 |

**Table S17.** The top 10 significantly enriched GO terms for the differential expressed genes (DEGs) in the comparison of ovarian and endometrial mRNAs in European mouflon based on the *de novo* transcriptome

| GO Term | GO number | Cluster genes | Total genes | Percentage  (%) | *P*-value |
| --- | --- | --- | --- | --- | --- |
| **Biological process** | | | | | |
| cellular process | GO:0009987 | 4901 | 6090 | 80.48 | 8.91E-93 |
| Biological process | GO:0008150 | 5499 | 6090 | 90.30 | 7.75E-92 |
| cellular metabolic process | GO:0044237 | 3225 | 6090 | 52.96 | 3.50E-76 |
| organic substance metabolic process | GO:0071704 | 3373 | 6090 | 55.39 | 1.26E-75 |
| metabolic process | GO:0008152 | 3490 | 6090 | 57.31 | 4.13E-75 |
| primary metabolic process | GO:0044238 | 3227 | 6090 | 52.99 | 1.11E-72 |
| cellular component organization or biogenesis | GO:0071840 | 2171 | 6090 | 35.65 | 1.82E-62 |
| cellular component organization | GO:0016043 | 2094 | 6090 | 34.38 | 2.00E-57 |
| macromolecule metabolic process | GO:0043170 | 2753 | 6090 | 45.21 | 9.61E-53 |
| detection of chemical stimulus involved in sensory perception | GO:0050907 | 3 | 6090 | 0.49 | 3.19E-51 |
| **Cellular process** | | | | | |
| intracellular part | GO:0044424 | 4978 | 6090 | 81.74 | 5.36E-172 |
| intracellular | GO:0005622 | 5051 | 6090 | 82.94 | 2.62E-165 |
| organelle | GO:0043226 | 4744 | 6090 | 77.90 | 1.27E-154 |
| intracellular organelle | GO:0043229 | 4438 | 6090 | 72.87 | 5.15E-142 |
| cytoplasm | GO:0005737 | 4087 | 6090 | 67.11 | 4.89E-140 |
| membrane-bounded organelle | GO:0043227 | 4421 | 6090 | 72.59 | 8.52E-130 |
| intracellular membrane-bounded organelle | GO:0043231 | 4084 | 6090 | 67.06 | 1.80E-122 |
| intracellular organelle part | GO:0044446 | 3221 | 6090 | 52.89 | 2.60E-109 |
| organelle part | GO:0044422 | 3273 | 6090 | 53.74 | 1.57E-108 |
| cell part | GO:0044464 | 5380 | 6090 | 88.34 | 9.06E-106 |
| **Molecular function** | | | | | |
| binding | GO:0005488 | 4989 | 6090 | 81.82 | 3.42E-123 |
| protein binding | GO:0005515 | 4010 | 6090 | 65.85 | 2.49E-107 |
| Molecular function | GO:0003674 | 5519 | 6090 | 90.62 | 2.16E-86 |
| catalytic activity | GO:0003824 | 2267 | 6090 | 37.22 | 7.70E-46 |
| G-protein coupled receptor activity | GO:0004930 | 86 | 6090 | 1.41 | 7.24E-33 |
| heterocyclic compound binding | GO:1901363 | 2169 | 6090 | 35.62 | 1.16E-32 |
| organic cyclic compound binding | GO:0097159 | 2194 | 6090 | 36.03 | 1.50E-32 |
| transferase activity | GO:0016740 | 994 | 6090 | 16.32 | 8.61E-24 |
| RNA binding | GO:0003723 | 702 | 6090 | 11.53 | 5.01E-22 |
| small molecule binding | GO:0036094 | 1028 | 6090 | 16.88 | 1.29E-21 |

**Table S18.** The top enriched pathways for the differential expressed genes (DEGs) in the comparison of ovarian and endometrial mRNAs in European mouflon based on the sheep reference genome Oar v.4.0 and the *de novo* transcriptome

| Pathway | Cluster genes | Total genes | Percentage | *P*-value |
| --- | --- | --- | --- | --- |
| **with sheep reference genome(Oarv.4.0)** | | | | |
| PI3K-Akt signaling pathway | 71 | 1949 | 3.64 | 1.02E-10 |
| ECM-receptor interaction | 29 | 1949 | 1.49 | 2.43E-09 |
| Focal adhesion | 46 | 1949 | 2.36 | 2.98E-08 |
| Pathways in cancer | 66 | 1949 | 3.39 | 1.08E-06 |
| Rap1 signaling pathway | 40 | 1949 | 2.05 | 7.44E-06 |
| Axon guidance | 28 | 1949 | 1.44 | 1.20E-05 |
| Protein digestion and absorption | 28 | 1949 | 1.44 | 2.22E-05 |
| Cell adhesion molecules (CAMs) | 32 | 1949 | 1.64 | 4.26E-05 |
| Vascular smooth muscle contraction | 26 | 1949 | 1.33 | 1.11E-04 |
| Calcium signaling pathway | 33 | 1949 | 1.69 | 1.74E-04 |
| ***de novo*** | | | | |
| Integrin signalling pathway | 91 | 6090 | 1.49% | 1.30E-03 |
| CCKR signaling map | 82 | 6090 | 1.35% | 3.50E-03 |
| TGF-beta signaling pathway | 55 | 6090 | 0.90% | 3.76E-03 |

**Table S19. GO annotation of the predicted target genes for 23 Finnsheep ovarian down-regulated miRNAs**

| **GO term** | **GO number** | **Cluster genes** | **Total genes** | **Percentage**  **(%)** | ***P*-value** |
| --- | --- | --- | --- | --- | --- |
| **Biological process** | | | | | |
| RNA splicing | GO:0008380 | 9 | 64 | 14.06 | 7.41E-05 |
| multi-organism process | GO:0051704 | 20 | 64 | 31.25 | 2.69E-04 |
| mRNA metabolic process | GO:0016071 | 10 | 64 | 15.63 | 5.22E-04 |
| RNA splicing, via transesterification reactions | GO:0000375 | 7 | 64 | 10.94 | 6.63E-04 |
| response to organic cyclic compound | GO:0014070 | 11 | 64 | 17.19 | 0.001123 |
| mRNA processing | GO:0006397 | 8 | 64 | 12.50 | 0.001264 |
| multi-organism reproductive process | GO:0044703 | 11 | 64 | 17.19 | 0.001435 |
| anatomical structure development | GO:0048856 | 31 | 64 | 48.44 | 0.002735 |
| single-organism developmental process | GO:0044767 | 31 | 64 | 48.44 | 0.002752 |
| RNA splicing, via transesterification reactions with bulged adenosine as nucleophile | GO:0000377 | 6 | 64 | 9.38 | 0.003953 |
| mRNA splicing, via spliceosome | GO:0000398 | 6 | 64 | 9.38 | 0.003953 |
| phosphatidylinositol-mediated signaling | GO:0048015 | 5 | 64 | 7.81 | 0.003971 |
| inositol lipid-mediated signaling | GO:0048017 | 5 | 64 | 7.81 | 0.004206 |
| RNA processing | GO:0006396 | 10 | 64 | 15.63 | 0.004239 |
| development of primary female sexual characteristics | GO:0046545 | 4 | 64 | 6.25 | 0.004403 |
| developmental process | GO:0032502 | 31 | 64 | 48.44 | 0.004429 |
| rhythmic process | GO:0048511 | 6 | 64 | 9.38 | 0.004731 |
| organophosphate metabolic process | GO:0019637 | 11 | 64 | 17.19 | 0.004887 |
| single-multicellular organism process | GO:0044707 | 32 | 64 | 50.00 | 0.005076 |
| multicellular organismal reproductive process | GO:0048609 | 9 | 64 | 14.06 | 0.005821 |
| multicellular organism reproduction | GO:0032504 | 9 | 64 | 14.06 | 0.006226 |
| female sex differentiation | GO:0046660 | 4 | 64 | 6.25 | 0.006477 |
| positive regulation of biological process | GO:0048518 | 29 | 64 | 45.31 | 0.007318 |
| reproductive process | GO:0022414 | 12 | 64 | 18.75 | 0.007615 |
| cellular response to organic cyclic compound | GO:0071407 | 7 | 64 | 10.94 | 0.009635 |
| multicellular organism development | GO:0007275 | 27 | 64 | 42.19 | 0.009868 |
| multicellular organismal process | GO:0032501 | 35 | 64 | 54.69 | 0.010379 |
| response to steroid hormone | GO:0048545 | 6 | 64 | 9.38 | 0.010761 |
| regulation of cell communication | GO:0010646 | 19 | 64 | 29.69 | 0.013277 |
| regulation of phosphatidylinositol 3-kinase signaling | GO:0014066 | 4 | 64 | 6.25 | 0.013631 |
| nucleobase-containing compound metabolic process | GO:0006139 | 30 | 64 | 46.88 | 0.014162 |
| regulation of signaling | GO:0023051 | 19 | 64 | 29.69 | 0.015617 |
| system development | GO:0048731 | 24 | 64 | 37.50 | 0.015928 |
| response to endogenous stimulus | GO:0009719 | 12 | 64 | 18.75 | 0.017734 |
| viral process | GO:0016032 | 9 | 64 | 14.06 | 0.0191 |
| heterocycle metabolic process | GO:0046483 | 30 | 64 | 46.88 | 0.019259 |
| multi-organism cellular process | GO:0044764 | 9 | 64 | 14.06 | 0.019858 |
| regulation of response to stimulus | GO:0048583 | 21 | 64 | 32.81 | 0.020288 |
| cellular aromatic compound metabolic process | GO:0006725 | 30 | 64 | 46.88 | 0.02148 |
| symbiosis, encompassing mutualism through parasitism | GO:0044403 | 9 | 64 | 14.06 | 0.022741 |
| interspecies interaction between organisms | GO:0044419 | 9 | 64 | 14.06 | 0.022741 |
| negative regulation of gonadotropin secretion | GO:0032277 | 2 | 64 | 3.13 | 0.024342 |
| positive regulation of cellular process | GO:0048522 | 25 | 64 | 39.06 | 0.026731 |
| female pregnancy | GO:0007565 | 4 | 64 | 6.25 | 0.028021 |
| positive regulation of cell proliferation | GO:0008284 | 8 | 64 | 12.50 | 0.028604 |
| nucleotide metabolic process | GO:0009117 | 7 | 64 | 10.94 | 0.029119 |
| response to lipid | GO:0033993 | 8 | 64 | 12.50 | 0.030393 |
| nucleoside phosphate metabolic process | GO:0006753 | 7 | 64 | 10.94 | 0.030624 |
| response to organic substance | GO:0010033 | 17 | 64 | 26.56 | 0.031061 |
| organic cyclic compound metabolic process | GO:1901360 | 30 | 64 | 46.88 | 0.032563 |
| cellular response to lipid | GO:0071396 | 6 | 64 | 9.38 | 0.032894 |
| secretion | GO:0046903 | 9 | 64 | 14.06 | 0.037278 |
| regulation of gonadotropin secretion | GO:0032276 | 2 | 64 | 3.13 | 0.037989 |
| gonad development | GO:0008406 | 4 | 64 | 6.25 | 0.038941 |
| ribonucleotide metabolic process | GO:0009259 | 6 | 64 | 9.38 | 0.039451 |
| multi-multicellular organism process | GO:0044706 | 4 | 64 | 6.25 | 0.039859 |
| development of primary sexual characteristics | GO:0045137 | 4 | 64 | 6.25 | 0.041257 |
| nucleobase-containing small molecule metabolic process | GO:0055086 | 7 | 64 | 10.94 | 0.041904 |
| regulation of cell proliferation | GO:0042127 | 11 | 64 | 17.19 | 0.042619 |
| ribose phosphate metabolic process | GO:0019693 | 6 | 64 | 9.38 | 0.043293 |
| pyruvate metabolic process | GO:0006090 | 3 | 64 | 4.69 | 0.045678 |
| ribonucleoside metabolic process | GO:0009119 | 5 | 64 | 7.81 | 0.046192 |
| positive regulation of cytokine production | GO:0001819 | 5 | 64 | 7.81 | 0.046557 |
| protein folding | GO:0006457 | 4 | 64 | 6.25 | 0.046592 |
| regulation of signal transduction | GO:0009966 | 16 | 64 | 25.00 | 0.046883 |
| **Cellular component** |  | | | | |
| nuclear speck | GO:0016607 | 6 | 64 | 9.38 | 5.60E-04 |
| nuclear body | GO:0016604 | 6 | 64 | 9.38 | 0.006963 |
| nucleoplasm | GO:0005654 | 19 | 64 | 29.69 | 0.007667 |
| extracellular space | GO:0005615 | 11 | 64 | 17.19 | 0.020793 |
| spliceosomal complex | GO:0005681 | 4 | 64 | 6.25 | 0.022122 |
| organelle lumen | GO:0043233 | 23 | 64 | 35.94 | 0.024621 |
| membrane-enclosed lumen | GO:0031974 | 23 | 64 | 35.94 | 0.028281 |
| anchoring junction | GO:0070161 | 7 | 64 | 10.94 | 0.03119 |
| nucleoplasm part | GO:0044451 | 7 | 64 | 10.94 | 0.037054 |
| catalytic step 2 spliceosome | GO:0071013 | 3 | 64 | 4.69 | 0.038057 |
| intracellular organelle lumen | GO:0070013 | 22 | 64 | 34.38 | 0.038829 |
| macromolecular complex | GO:0032991 | 24 | 64 | 37.50 | 0.03931 |
| nuclear lumen | GO:0031981 | 19 | 64 | 29.69 | 0.0435 |
| protein complex | GO:0043234 | 21 | 64 | 32.81 | 0.043932 |
| intracellular ribonucleoprotein complex | GO:0030529 | 7 | 64 | 10.94 | 0.047117 |
| ribonucleoprotein complex | GO:1990904 | 7 | 64 | 10.94 | 0.047358 |
| **Molecular function** | | | | | |
| poly(A) RNA binding | GO:0044822 | 15 | 64 | 23.44 | 3.20E-05 |
| RNA binding | GO:0003723 | 17 | 64 | 26.56 | 8.02E-05 |
| receptor binding | GO:0005102 | 13 | 64 | 20.31 | 0.003069 |
| transferase activity, transferring pentosyl groups | GO:0016763 | 3 | 64 | 4.69 | 0.016946 |
| heterocyclic compound binding | GO:1901363 | 29 | 64 | 45.31 | 0.022469 |
| catalytic activity | GO:0003824 | 29 | 64 | 45.31 | 0.024991 |
| organic cyclic compound binding | GO:0097159 | 29 | 64 | 45.31 | 0.026955 |
| cytokine activity | GO:0005125 | 4 | 64 | 6.25 | 0.039072 |
| oxidoreductase activity, acting on a sulfur group of donors, NAD(P) as acceptor | GO:0016668 | 2 | 64 | 3.13 | 0.046266 |

**Table S20. GO annotation KEGG pathways of the predicted target genes for 30 European mouflon ovarian down-regulated miRNAs**

| **GO term** | **GO number** | **Cluster**  **genes** | **Total genes** | **Percentage**  **(%)** | ***P*-value** |
| --- | --- | --- | --- | --- | --- |
| **Biological process** | | | | | |
| aorta development | GO:0035904 | 3 | 159 | 1.88 | 0.027883 |
| transmembrane receptor protein serine/threonine kinase signaling pathway | GO:0007178 | 6 | 159 | 3.77 | 0.030693 |
| cellular response to chemical stimulus | GO:0070887 | 22 | 159 | 1.38 | 0.042969 |
| cellular response to growth factor stimulus | GO:0071363 | 8 | 159 | 5.03 | 0.047575 |
| **Cellular component** | | | | | |
| membrane-bounded organelle | GO:0043227 | 78 | 159 | 49.00 | 4.91E-04 |
| cytoplasm | GO:0005737 | 69 | 159 | 43.34 | 0.003439 |
| organelle | GO:0043226 | 79 | 159 | 49.62 | 0.005986 |
| cytoplasmic part | GO:0044444 | 54 | 159 | 33.92 | 0.009675 |
| intracellular | GO:0005622 | 81 | 159 | 50.88 | 0.035598 |
| intracellular membrane-bounded organelle | GO:0043231 | 66 | 159 | 41.46 | 0.039156 |
| vesicle membrane | GO:0012506 | 7 | 159 | 4.40 | 0.045204 |
| **Molecular function** | | | | | |
| catalytic activity | GO:0003824 | 42 | 159 | 26.38 | 0.022163 |
| growth factor binding | GO:0019838 | 4 | 159 | 2.51 | 0.029305 |
| lipid kinase activity | GO:0001727 | 2 | 159 | 1.26 | 0.030877 |
| macromolecular complex binding | GO:0044877 | 13 | 159 | 8.17 | 0.038105 |
| hydrolase activity | GO:0016787 | 21 | 159 | 13.19 | 0.04469 |
| **KEGG pathway** | | | | | |
| Metabolic pathways | hsa01100 | 13 | 159 | 8.17 | 0.027532 |

**Table S21.** GO annotation of the predicted target genes for 4 Finnsheep endometrial down-regulated miRNAs

| **GO term** | **GO number** | **Cluster**  **genes** | **Total**  **genes** | **Percentage**  **(%)** | ***P*-value** |
| --- | --- | --- | --- | --- | --- |
| **Biological process** | | | | | |
| coenzyme biosynthetic process | GO:0009108 | 4 | 88 | 4.54 | 0.001565 |
| cofactor biosynthetic process | GO:0051188 | 4 | 88 | 4.54 | 0.004399 |
| ameboidal-type cell migration | GO:0001667 | 5 | 88 | 5.67 | 0.008926 |
| blood vessel endothelial cell migration | GO:0043534 | 3 | 88 | 3.40 | 0.012152 |
| regulation of multi-organism process | GO:0043900 | 5 | 88 | 5.67 | 0.014665 |
| coenzyme metabolic process | GO:0006732 | 4 | 88 | 4.54 | 0.019911 |
| regulation of endothelial cell migration | GO:0010594 | 3 | 88 | 3.40 | 0.033499 |
| cofactor metabolic process | GO:0051186 | 4 | 88 | 4.54 | 0.035065 |
| regulation of symbiosis, encompassing mutualism through parasitism | GO:0043903 | 4 | 88 | 4.54 | 0.03865 |
| symbiosis, encompassing mutualism through parasitism | GO:0044403 | 5 | 88 | 5.67 | 0.039745 |
| interspecies interaction between organisms | GO:0044419 | 5 | 88 | 5.67 | 0.039745 |
| organophosphate metabolic process | GO:0019637 | 6 | 88 | 6.81 | 0.043989 |
| pyridine-containing compound biosynthetic process | GO:0072525 | 2 | 88 | 2.27 | 0.046468 |
| cell migration | GO:0016477 | 7 | 88 | 7.94 | 0.047749 |
| **Cellular component** | | | | | |
| organelle | GO:0043226 | 35 | 88 | 39.72 | 0.014401 |
| membrane-bounded organelle | GO:0043227 | 33 | 88 | 37.45 | 0.015629 |
| cytoplasm | GO:0005737 | 28 | 88 | 31.77 | 0.03077 |
| cytoplasmic part | GO:0044444 | 21 | 88 | 23.83 | 0.033936 |
| **Molecular function** | | | | | |
| catalytic activity | GO:0003824 | 24 | 88 | 27.24 | 0.014726 |
| phosphoric ester hydrolase activity | GO:0042578 | 5 | 88 | 5.67 | 0.017956 |
| small protein activating enzyme activity | GO:0008641 | 2 | 88 | 2.27 | 0.033914 |

**Table S22.** GO annotation of the predicted target genes for 1 European mouflon endometrial down-regulated miRNAs

| **GO term** | **GO number** | **Cluster genes** | **Total**  **genes** | **Percentage**  **(%)** | ***P*-value** |
| --- | --- | --- | --- | --- | --- |
| **Biological process** | | | | | |
| regulation of epithelial cell proliferation | GO:0050678 | 3 | 14 | 21.44 | 0.006296 |
| response to abiotic stimulus | GO:0009628 | 4 | 14 | 28,59 | 0.006664 |
| epithelial cell proliferation | GO:0050673 | 3 | 14 | 21.44 | 0.008348 |
| cardiac epithelial to mesenchymal transition | GO:0060317 | 2 | 14 | 14.30 | 0.012854 |
| blood vessel morphogenesis | GO:0048514 | 3 | 14 | 21.44 | 0.016254 |
| regulation of double-strand break repair | GO:2000779 | 2 | 14 | 14.30 | 0.017381 |
| heart development | GO:0007507 | 3 | 14 | 21.44 | 0.017536 |
| regulation of cell proliferation | GO:0042127 | 4 | 14 | 28,59 | 0.020752 |
| blood vessel development | GO:0001568 | 3 | 14 | 21.44 | 0.021997 |
| tube development | GO:0035295 | 3 | 14 | 21.44 | 0.022286 |
| regulation of cellular response to stress | GO:0080135 | 3 | 14 | 21.44 | 0.022358 |
| vasculature development | GO:0001944 | 3 | 14 | 21.44 | 0.024505 |
| cellular response to growth factor stimulus | GO:0071363 | 3 | 14 | 21.44 | 0.024505 |
| tissue morphogenesis | GO:0048729 | 3 | 14 | 21.44 | 0.025418 |
| response to growth factor | GO:0070848 | 3 | 14 | 21.44 | 0.026345 |
| outflow tract morphogenesis | GO:0003151 | 2 | 14 | 14.30 | 0.028823 |
| regulation of DNA repair | GO:0006282 | 2 | 14 | 14.30 | 0.034097 |
| palate development | GO:0060021 | 2 | 14 | 14.30 | 0.034906 |
| regulation of cellular component movement | GO:0051270 | 3 | 14 | 21.44 | 0.040769 |
| epithelial to mesenchymal transition | GO:0001837 | 2 | 14 | 14.30 | 0.042564 |
| regulation of striated muscle tissue development | GO:0016202 | 2 | 14 | 14.30 | 0.047374 |
| negative regulation of epithelial cell proliferation | GO:0050680 | 2 | 14 | 14.30 | 0.048174 |
| regulation of muscle organ development | GO:0048634 | 2 | 14 | 14.30 | 0.048174 |
| regulation of muscle tissue development | GO:1901861 | 2 | 14 | 14.30 | 0.048573 |
| cardiocyte differentiation | GO:0035051 | 2 | 14 | 14.30 | 0.049771 |
| **Cellular component** | | | | | |
| proteinaceous extracellular matrix | GO:0005578 | 3 | 14 | 21.44 | 0.007694 |
| extracellular matrix | GO:0031012 | 3 | 14 | 21.44 | 0.016089 |
| basement membrane | GO:0005604 | 2 | 14 | 14.30 | 0.03593 |
| extracellular matrix component | GO:0044420 | 2 | 14 | 14.30 | 0.04815 |
| **Molecular function** | | | | | |
| protein homodimerization activity | GO:0042803 | 3 | 14 | 21.44 | 0.024944 |
| carbohydrate derivative binding | GO:0097367 | 4 | 14 | 28.59 | 0.034217 |

**Table S23.** Validation of miRNA-target gene pairs in previous publications

| miRNA | Target genes | References |
| --- | --- | --- |
| ***miR-197-3p*** | *CD82* | Dai *et al*. 2014 |
| *let-7c-5p* | *SALL4* | Melton *et al*. 2010 |
| *miR-22-3p* | *LGALS1* | White *et al*. 2014 |
| *miR-148a-3p* | *SERPINE1* | Tseng *et al*. 2011 |
| ***miR-143-3p*** | *SERPINE1* | Villadsen *et al*. 2012 |
| *miR-30b-5p* | *SERPINE1* | Zhu *et al*. 2014 |
| *miR-140-3p* | *SLC30A3* | Karginov *et al*. 2013 |
| *miR-615-3p* | *NCLN* | Helwak *et al*. 2013 |
| *miR-197-3p* | *PLIN3* | Karginov *et al*. 2013 |
| ***miR-432-5p*** | *PRKAB1* | Karginov *et al*. 2013 |
| *miR-423-3p* | *CENPM* | Xue *et al*. 2013 |
| ***miR-331-3p*** | *KAT2A, NCLN* | Helwak *et al*. 2013 |
| *miR-485-5p* | *ZNF500, NUP85* | Whisnant *et al*. 2013; Karginov *et al*. 2013 |
| *let-7e-5p* | *NME4, RAB40C, SNX17, DUSP1* | Helwak *et al*. 2013; Whisnant *et al*. 2013; Skalsky *et al*. 2012; Kishore *et al*. 2011 |
| ***let-7c-5p*** | *NME4, RAB40C, SNX17, DUSP1* | Helwak *et al*. 2013; Whisnant *et al*. 2013; Skalsky *et al*. 2012; Kishore *et al*. 2011 |
| ***miR-93-5p*** | *ATP5SL, PPP6R2, PPM1H, TBC1D17, CDIPT* | Hafner *et al*. 2010; Helwak *et al*. 2013; Riley *et al*. 2012; Chi *et al*. 2009 |
| ***miR-101-3p*** | *TSPAN12, SLC35F5, PIAS1* | Hafner *et al*. 2010; Whisnant *et al*. 2013; Kishore *et al*. 2011; Memczak *et al*. 2013; Karginov *et al*. 2013 |
| ***miR-374a-5p*** | *UBE3A, PANK3* | Kishore *et al*. 2011; Hafner *et al*. 2010 |
| ***miR-148a-3p*** | *AGO2, SOS2* | Kishore *et al*. 2011; Farazi *et al*. 2014; Karginov *et al*. 2013 |
| ***miR-335-5p*** | *FLT3, IFT81* | Tavazoie *et al*. 2008 |
| *miR-9-5p* | *PANK3, CDK13, TWISTNB, UMPS* | Chi *et al*. 2009; Grimson *et al*. 2007; Hafner *et al*. 2010; Karginov *et al*. 2013 |
| *miR-340-5p* | *PANK3, SPP1, PIAS1,TARDBP* | Hafner *et al*. 2010; Zhang *et al*. 2014; Loeb *et al*. 2012; Kishore *et al*. 2011 |
| ***miR-21-5p*** | *RP2, PAG1, AGO2, FAM136A* | Gabriely *et al*. 2008; Riley *et al*. 2012; Hafner *et al*. 2010; Memczak *et al*. 2013; Farazi *et al*. 2014; Whisnant *et al*. 2013 |
| ***miR-27a-3p*** | *FAM136A, TARDBP* | Whisnant *et al*. 2013 |
| *miR-30e-5p* | *PNN, PANK3, SERPINE1* | Chi *et al*. 2009; Lipchina *et al*. 2011; Loeb *et al*. 2012 |
| ***miR-374b-5p*** | *UBE3A, PANK3* | Kishore *et al*. 2011; Hafner *et al*. 2010 |
| ***miR-30b-5p*** | *PNN, SERPINE1* | Hafner *et al*. 2010; Lipchina *et al*. 2011; Loeb *et al*. 2012 |
| ***miR-98-5p*** | *FKBP3* | Gennarino *et al*. 2009 |
| ***miR-27b-3p*** | *FAM136A, TARDBP* | Whisnant *et al*. 2013 |
| ***miR-10b-5p*** | *NUFIP2, GSS* | Helwak *et al*. 2013; Lipchina *et al*. 2011; Leung *et al*. 2011 |
| ***miR-92a-3p*** | *USP31, VPS4B, NUFIP2, SGPP1, JOSD1* | Helwak *et al*. 2013; Kishore *et al*. 2011; Riley *et al*. 2012 |

The miRNAs with the IDs in bold are without the suffixes of 3p or 5p in our study, but the two types (e.g. *miR-197-3p* and *miR-197*) have the same sequences.
